# Analyzing gut microbiota composition in individual *Anopheles* mosquitoes after experimental treatment

**DOI:** 10.1016/j.isci.2021.103416

**Published:** 2021-11-09

**Authors:** Aminata Fofana, Mathilde Gendrin, Ottavia Romoli, G. Armel Bienvenu Yarbanga, Georges Anicet Ouédraogo, Rakiswende Serge Yerbanga, Jean-Bosco Ouédraogo

**Affiliations:** 1 Institut de Recherche en Sciences de la Santé, Bobo Dioulasso, Burkina Faso; 2 Université Nazi Boni, Bobo-Dioulasso 1091, Burkina Faso; 3 Microbiota of Insect Vectors Group, Institut Pasteur de la Guyane, 97306 Cayenne, French Guiana; 4 Institut Pasteur, Université de Paris, Department of Insect Vectors, 75015 Paris, France; 5 Institut des Sciences et Techniques, 2779 Bobo Dioulasso, Burkina Faso

**Keywords:** Entomology, Microbiome

## Abstract

The microbiota of *Anopheles* mosquitoes influences malaria transmission. Antibiotics ingested during a blood meal impact the mosquito microbiome and malaria transmission, with substantial differences between drugs. Here, we assessed if amoxicillin affects the gut mosquito microbiota*.* We collected *Anopheles* larvae in Burkina Faso, kept them in semi-field conditions, and offered a blood meal to adult females. We tested the impact of blood supplementation with two alternative amoxicillin preparations on microbiota composition, determined by high-throughput sequencing in individual gut samples. Our analysis detected four major genera, *Elizabethkingia*, *Wigglesworthia*, *Asaia,* and *Serratia*. The antibiotic treatment significantly affected overall microbiota composition, with a specific decrease in the relative abundance of *Elizabethkingia* and *Asaia* during blood digestion. Besides its interest on the influence of amoxicillin on the mosquito microbiota, our study proposes a thorough approach to report negative-control data of high-throughput sequencing studies on samples with a reduced microbial load.

## Highlights

• We sequenced bacterial 16S in mosquitoes collected as larvae in Burkina Faso
• *Elizabethkingia*, *Wigglesworthia*, *Asaia*, and *Serratia* dominate the gut microbiota
• Amoxicillin treatment and potentially gametocyte presence influence the microbiota
• We set up methods to consider mock controls after sequencing low-biomass samples

## Graphical abstract

**Figure:**
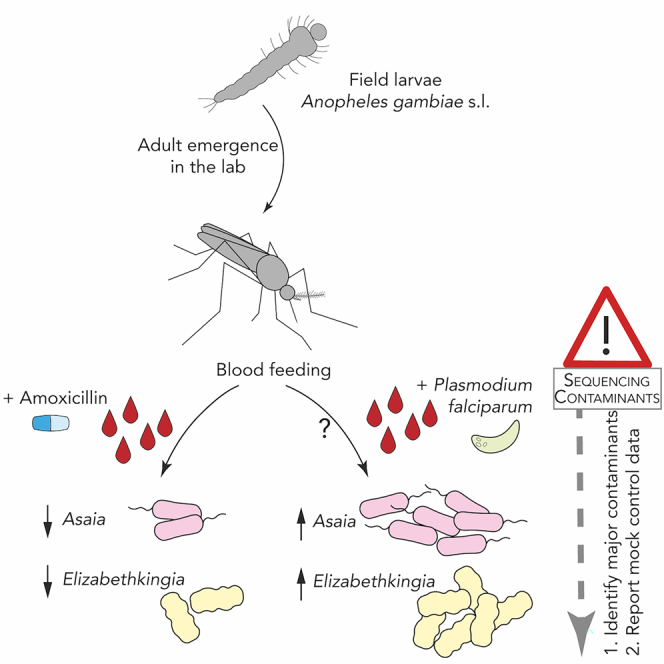

## Introduction

Malaria remains one of the most devastating diseases. The World Health Organization (WHO) estimated 229 million malaria cases in 2019 with 409,000 deaths (World Health Organization, 2020), and 94% of the cases was recorded in the WHO African region (World Health Organization, 2020). *Plasmodium falciparum* is the etiological agent in Sub-Saharan Africa and *Anopheles gambiae s.l.* its major vector. Malaria control requires the coordination of different strategies due to the lack of an effective vaccine and the emerging resistance of parasites to drugs and of vectors to insecticides. Alternative novel strategies are being developed to limit disease transmission via suppression of vector populations or their replacement with less efficient vectors. These approaches are notably based on the release of sterile males, the genetic manipulation of the mosquito and/or the modification of its microbiota.

The ability of mosquitoes to transmit pathogens is influenced by the bacterial microbiota. Most studies investigating these microbiota-pathogen interactions used antibiotic treatments to eliminate the microbiota rather than to monitor the impact of physiological exposure (Dong et al., 2009; Guégan et al., 2018; Kalappa et al., 2018; Martínez-de la Puente et al., 2021; Meister et al., 2009). Such treatments were provided via the sugar meal at nontherapeutic concentrations but have been shown to cause dysbiosis rather than a complete elimination of the microbiota (Hughes et al., 2014). Other studies investigated the effect of antibiotic exposure during blood feeding and showed that mosquito gut bacteria are affected by certain antibiotics commonly used to treat infectious diseases. Of note, a cocktail of penicillin and streptomycin added to the blood meal of mosquitoes at therapeutic concentrations significantly decreased bacterial growth in the gut and increased mosquito survival, fecundity, and permissiveness to *Plasmodium* infection (Gendrin et al., 2015). The effect of three antibiotics (azithromycin, doxycycline, and co-trimoxazole) in mosquitoes was subsequently investigated to test the combined direct and microbiota-mediated impacts of these three drugs, which all have antiparasitic effects in addition to their antibacterial activity (Gendrin et al., 2016). Similar to the impact of penicillin-streptomycin, an increase in *P. falciparum* infection load was observed when exposed to doxycycline at high dose. On the contrary, a negative effect on both bacterial load and *Plasmodium* infection intensity was observed with a co-trimoxazole or azithromycin treatment, suggesting a direct negative impact of these antimicrobials on mosquito stages of parasite development. Together, these studies indicate that different antimicrobial drugs have variable effects on the mosquito, underlying the need for their characterization with a wider number of antibiotics. Besides the direct negative impact of drugs on *Plasmodium*, such variations may be explained by differences in the efficiency of these drugs to clear bacteria of the mosquito gut or by the presence of different antibiotic-resistant bacteria. In fact, several microorganisms with variable levels of antibiotic sensitivity could be isolated from mosquito larvae or adults (Coon et al., 2016; Hyde et al., 2019; Kämpfer et al., 2011). We have previously studied the antibiotic-resistance profiles of 21 bacterial strains belonging to 12 genera isolated from guts of *An. gambiae s.s.* in Burkina Faso. We found highly variable proportion of resistant strains between the 9 tested drugs, from 0% (8/8) for erythromycin to 76% (13/17) for ampicillin (Yerbanga et al., 2017). An amoxicillin/clavulanic acid cocktail showed intermediate growth inhibition efficiency, as 41% of our isolates (7/17) was resistant to this cocktail.

The impact of antibiotics on malaria transmission may be of importance in Sub-Saharan Africa, where mass-drug administration campaigns are organized to fight against neglected tropical diseases and where irrational prescriptions and self-medication with antibiotics are frequent (Ouedraogo et al., 2017). Recent studies on antibiotics showed that amoxicillin was the most frequently prescribed antibiotic (Ahiabu et al., 2016; Blaise Savadogo et al., 2014; Ojo et al., 2014). Therefore, we investigated whether this drug affects the mosquito microbiota composition. Considering the wide spread of antimicrobial resistance against β-lactam antibiotics, amoxicillin is typically provided with clavulanic acid, a β-lactamase inhibitor. In this study, we found that amoxicillin or a cocktail of amoxicillin and clavulanic acid affects the composition of the gut bacterial microbiota of *An. gambiae s.l.* females collected as larvae in natural breeding sites in Burkina Faso. As contaminant DNA and cross-contamination detected by metagenomic techniques can create confounding interpretation in the analysis of microbiome data particularly in low biomass samples (Dada et al., 2021; Eisenhofer et al., 2019), this study also led us to question the way we take negative controls into account when studying microbiota from individual mosquito guts. We propose that data from negative controls and from samples need to be reported together throughout such study.

## Results

### *Anopheles gambiae* s.l. collection

We collected *An. gambiae s.l.* larvae in Pè village and its surroundings during 3 consecutive collection campaigns in September, October, and December 2019, i.e. at the end of the rainy season, as mosquito numbers are higher (Table 1, Figure S1). We kept larvae in their breeding water until pupation and adults under standard insectary conditions and offered them a blood meal collected from *Plasmodium* gametocyte-infected donors (September, December) or from an uninfected donor (October). None of them had received any drug or herbal treatment during at least ten days prior to blood donation. In order to assess if amoxicillin can affect mosquito microbiota composition, we supplemented blood with amoxicillin, with a cocktail of amoxicillin and clavulanic acid, or with saline solution as a mock treatment. We determined the composition of the gut microbiota of 90 individuals via Miseq sequencing of the V3-V4 region of the bacterial *16S* gene. Samples were prepared from mosquitoes fed on gametocyte-infected or uninfected blood dissected 24 h and 72 h after blood feeding (see Table S1 for a complete sample description). Among these samples, *An. gambiae* was the most frequent with 54% (49/90) followed by *An. coluzzii* and *An. arabiensis* with 12% (11/90) and 18% (16/90), respectively. Sixteen percent (14/90) of the mosquitoes was not identified because of technical problems in molecular biology (Table 1).

**Table 1:** Description of the samples included in this analysis

Treatment/Date	Site^a^	Mosquito species			
		*An. gambiae*	*An. coluzzii*	*An. arabiensis*	ND^b^
Control					

09/2019	Pè	0	0	0	9
10/2019	Pè, Kari, Koumbia, Sebedougou	6	0	2	0
12/2019	Pè, Kari, Koumbia Sebedougou, Hounde	6	3	3	0

Amoxicillin					

09/2019	Pè	6	1	0	0
10/2019	Pè, Kari, Koumbia, Sebedougou	4	1	4	2
12/2019	Pè, Kari, Koumbia Sebedougou, Hounde	6	3	3	1

Amoxicillin + clavulanic acid					

09/2019	Pè	6	0	0	2
10/2019	Pè, Kari, Koumbia, Sebedougou	9	0	1	0
12/2019	Pè, Kari, Koumbia Sebedougou, Hounde	6	3	3	0
Total		49	11	16	14

a Locations of breeding sites are depicted on maps of Figure S1 based on (Base Nationale de Données Topographiques du Burkina Faso (BNDT), 2015a, 2015b; Openstreetmap contributors, n.d.).

b ND—nondetermined: samples for which PCR results did not allow any clear species identification.

### Discrimination between bacterial composition in samples and in negative controls

Detection and monitoring of contaminants are crucial when analyzing the bacteria composition of samples with low microbial biomass such as individual mosquito guts. In order to identify contaminants, each experimental replicate included a negative control consisting of a PBS aliquot, which was processed together with mosquito samples. Amplicon sequence variants (ASVs) found in negative controls might include contaminants from the environment, but also cross-contaminants coming from samples (Eisenhofer et al., 2019). Thus, the removal of all ASVs found in negative controls is a very conservative approach, which may lead to the elimination of sequences with biological relevance. Recently, a method to identify contaminants based on ASV prevalence in sequenced negative controls has been developed and can be implemented via the “decontam” R package (Davis et al., 2018). It is based on the observation that contaminants are found more often in negative controls than in true samples. We used this method with a 0.5 threshold, which classifies ASV as contaminants if present in a higher proportion of negative controls than of samples. Using this method, we identified 197 ASVs as potential contaminants (Table S2). However, this method did not suit very well our experimental setup, as only three negative controls were sequenced. Moreover, it does not account for cross-contaminations among samples. When sorting out major contaminant ASVs, we instead assumed that they would be enriched in negative controls compared with samples (average abundance in negative controls higher than in true samples) and present in a high proportion of the samples (at least 80%). We tested these assumptions using both raw read counts and relative abundance. Because our data generated a similar number of reads across samples and controls, the contaminant ASVs identified with read counts corresponded to those identified using relative abundance, except for one ASV that was identified only using raw read counts but was almost significant using relative abundance (Table S2). We decided to consider as contaminants only the six ASVs identified using relative abundance and we excluded them from all samples. Three of these six ASVs were also identified with the “decontam” method (Table S2). However, both our method and the “decontam” approach left some ASVs present in the negative controls. Hence, we first analyzed whether sequencing data were different in negative controls and in samples after removing the contaminants identified with these two methods.

The alpha diversity measured with both Chao and Shannon indexes seemed higher, albeit nonsignificantly, in negative controls than in samples whether contaminants were not removed or whether they were removed with both methods (Figures S2A–S2C, Wilcoxon test, no polishing: Chao1 index p = 0.76, Shannon index p = 0.35; “decontam”: Chao1 index p = 0.76, Shannon index p = 0.23; our method: Chao1 index p = 0.76, Shannon index p = 0.42; analysis performed on non-antibiotic treated samples). In line with previously published data on highly diluted samples (Biesbroek et al., 2012), these results suggest that negative controls have indeed a specific microbial composition and that they contain a high number of small abundance contaminants while guts are colonized by a less diverse microbiota. The statistical analysis on beta diversity indicated a significantly different Bray-Curtis dissimilarity between samples and negative controls after removing contaminants with our method (Figure S2F, PERMANOVA, F = 1.7, p = 0.045), whereas beta diversity was not significantly different when no ASV was removed (Figure S2D, PERMANOVA, F = 1.7, p = 0.09) or when ASVs were removed using the “decontam” method (Figure S2E, PERMANOVA, F = 1.5, *p* = 0.148). This suggests that major ASV identification with our method was the most appropriate approach for the analysis of our sequencing data.

Despite our efforts to identify potential contaminants, some midgut samples appeared close to extraction negative controls in the Principal Coordinates Analysis (PCoA) plot (Figure S2F). This observation was corroborated with Bray-Curtis dissimilarity indexes, as the average (±SEM) dissimilarity of the six samples closest to their corresponding PBS control was lower (0.64 ± 0.0074) than the average dissimilarity calculated between all samples and their PBS controls (0.84 ± 0.027, Wilcoxon test, p < 0.0001). Thus, these six individuals may carry a very small amount of bacterial DNA so that their composition is more difficult to discriminate from the controls, whereas other samples with a higher bacterial load may have driven the observed significant difference between samples and negative controls. Thus, we decided to report the results obtained on PBS negative samples throughout the whole analysis. We also report the unpolished table showing the taxonomy of all ASVs and their frequencies in each sample and negative control (Table S3).

### Replicate and/or donor infection status rather than mosquito species influence microbiota composition

**Figure 1:**
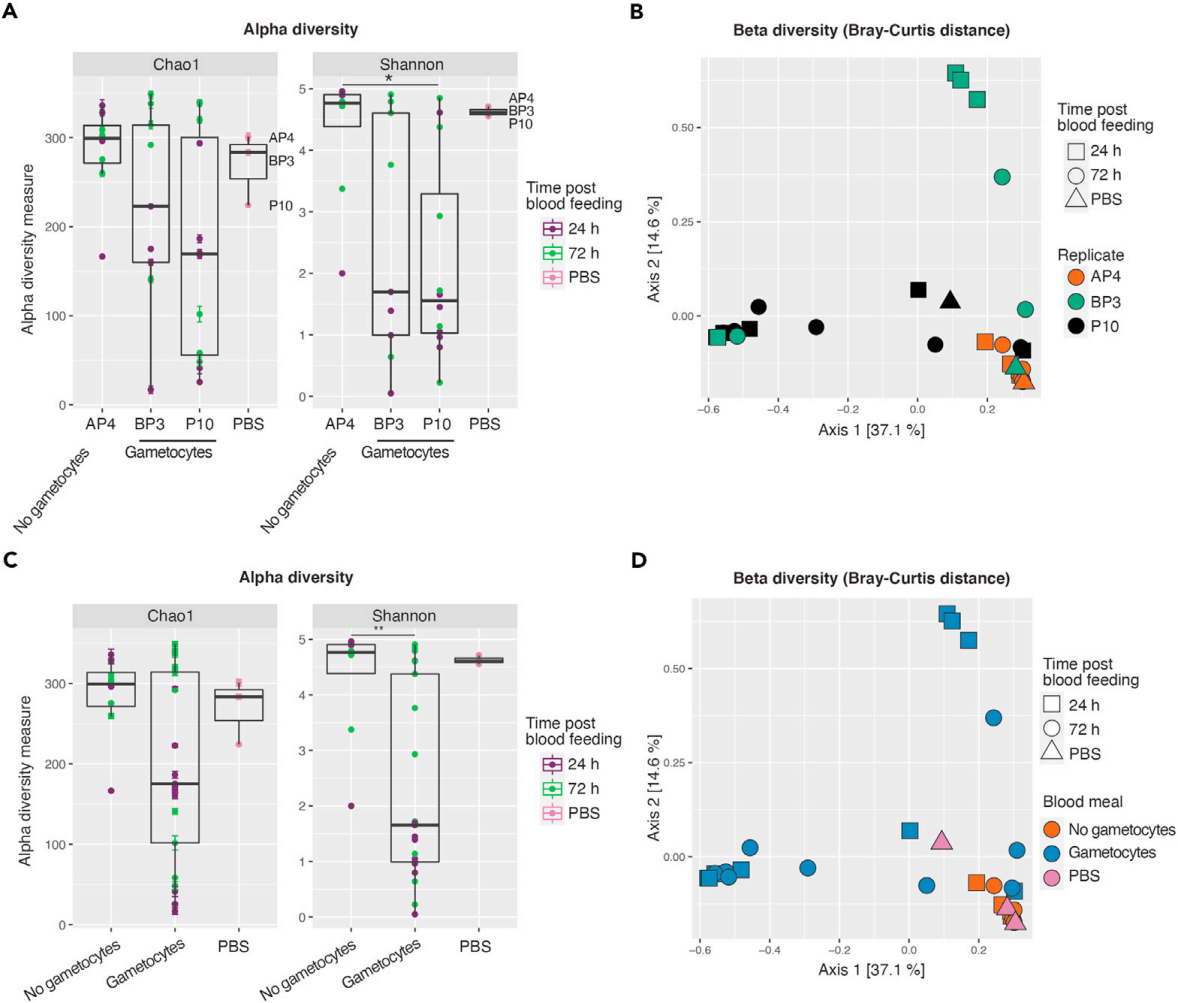
Replicate and/or donor infection status influence microbiota composition (A) Alpha diversity of gut bacterial microbiota of *An. gambiae s.l.* mosquitoes from different replicates (named by the code of blood donors) measured with Chao and Shannon indexes. Single points correspond to individual mosquito indexes. Color code corresponds to timing after blood feeding (purple: 24 h; green: 72 h; pink: extraction negative controls). Wilcoxon/Holm between replicates, Chao index: AP4 versus BP3 p = 1.00, AP4 versus P10 p = 0.81, BP3 versus P10 p = 1.00; Shannon index: AP4 versus BP3 p = 0.18, AP4 versus P10 p = 0.013, BP3 versus P10 p = 0.84. (B) Beta diversity of gut bacterial microbiota of *An. gambiae s.l.* mosquitoes represented by a Principal Coordinates Analysis (PCoA) plot of Bray-Curtis dissimilarity. Each point represents the microbial composition of a single mosquito. Mosquitoes from different replicates are highlighted with different colors (orange: AP4, no gametocytes; green: BP3, gametocytes; black: P10, gametocytes). The shape of each point indicates time after blood feeding (squares: 24 h; circles: 72 h; triangles: extraction negative controls; PERMANOVA on replicates, F = 3.5, p = 0.001). (C) Alpha diversity of gut bacterial microbiota of *An. gambiae s.l.* mosquitoes fed on mock-treated blood containing or not containing *P. falciparum* gametocytes. Plot parameters are as in (A) Wilcoxon/Holm, Chao index: infected versus uninfected p = 0.50; Shannon index: infected versus uninfected *p* = 0.0069. (D) Beta diversity of gut bacterial microbiota of *An. gambiae s.l.* mosquitoes represented by a PCoA plot of Bray-Curtis dissimilarity. Mosquitoes fed on blood containing or not containing *P. falciparum* gametocytes are highlighted with different colors (orange: no gametocytes; blue: gametocytes; pink: extraction negative controls; PERMANOVA, F = 3.1, p = 0.003). Point shapes are as in (B). Tests between time points (24 h versus 72 h) in alpha diversity (A and C): Wilcoxon/Holm, Chao index: p = 0.57; Shannon index: p = 0.74; in beta diversity (B and D): PERMANOVA, F = 1.4, p = 0.11. In A and C, the black horizontal line indicates the median, boxplot height indicates the upper and lower quartiles, whiskers indicate the maximum and minimum values. Error bars in Chao1 plots indicate standard errors. More data on statistics can be found in Table S4.

To identify the main factors driving the differences in the mosquito microbiota composition, we first evaluated whether bacterial species richness or relative abundance were related to replicate. Indeed, mosquitoes were collected at three different dates and fed with blood from three distinct donors, which may induce differences in microbiota composition. Only mock-treated samples were included in these analyses, to remove any confounding factor related to antibiotic exposure. The highest alpha diversity was observed in mosquitoes of the October replicate, fed on AP4 uninfected blood (Figure 1A, Wilcoxon/Holm, Chao index: AP4 versus BP3 p = 1.0, AP4 versus P10 p = 0.81, BP3 versus P10 p = 1.0; Shannon index: AP4 versus BP3 p = 0.18, AP4 versus P10 p = 0.013, BP3 versus P10 p = 0.84) and microbial compositions were also significantly different between replicates (Figure 1B, PERMANOVA, F = 3.5, p = 0.001). We also analyzed data after pooling results from both infected donors, to get some hints on potential effects of gametocyte carriage in the blood. Chao and Shannon indexes were lower in mosquitoes fed on blood containing *P. falciparum* gametocytes than on uninfected blood (Figure 1C, Wilcoxon/Holm test, Chao index: p = 0.50; Shannon index: p = 0.0069). The PCoA of Bray-Curtis dissimilarity indexes further showed a statistically significant distinction in the microbial composition of mosquitoes fed with *Plasmodium*-infected and uninfected blood (Figure 1D, PERMANOVA, F = 3.1, p = 0.003). However, the PCoA plots further divided mosquitoes fed on infected blood into two distinct groups. Thus, the observed differences in the microbiota composition can be related to the presence/absence of *P. falciparum* gametocytes in the blood or to replicate and/or donor effect.

Blood is digested in the midgut during around 48 h and then digestion remains are expelled as a fecal pellet together with the vast majority of the microbiota (Rodgers et al., 2017). We compared gut bacterial composition during and after digestion to assess whether some bacteria are discharged more efficiently than others in the fecal pellet (24 h and 72 h after blood feeding, respectively). Alpha diversity was slightly lower during digestion, albeit nonsignificantly (Figures 1A and 1C; Wilcoxon/Holm, Chao index: p = 0.23; Shannon index: p = 0.70). Bray-Curtis dissimilarity was nonsignificantly different, albeit with a too low p value to rule out any composition change (Figure 1B, PERMANOVA, F = 1.4, p = 0.11).

**Figure 2:**
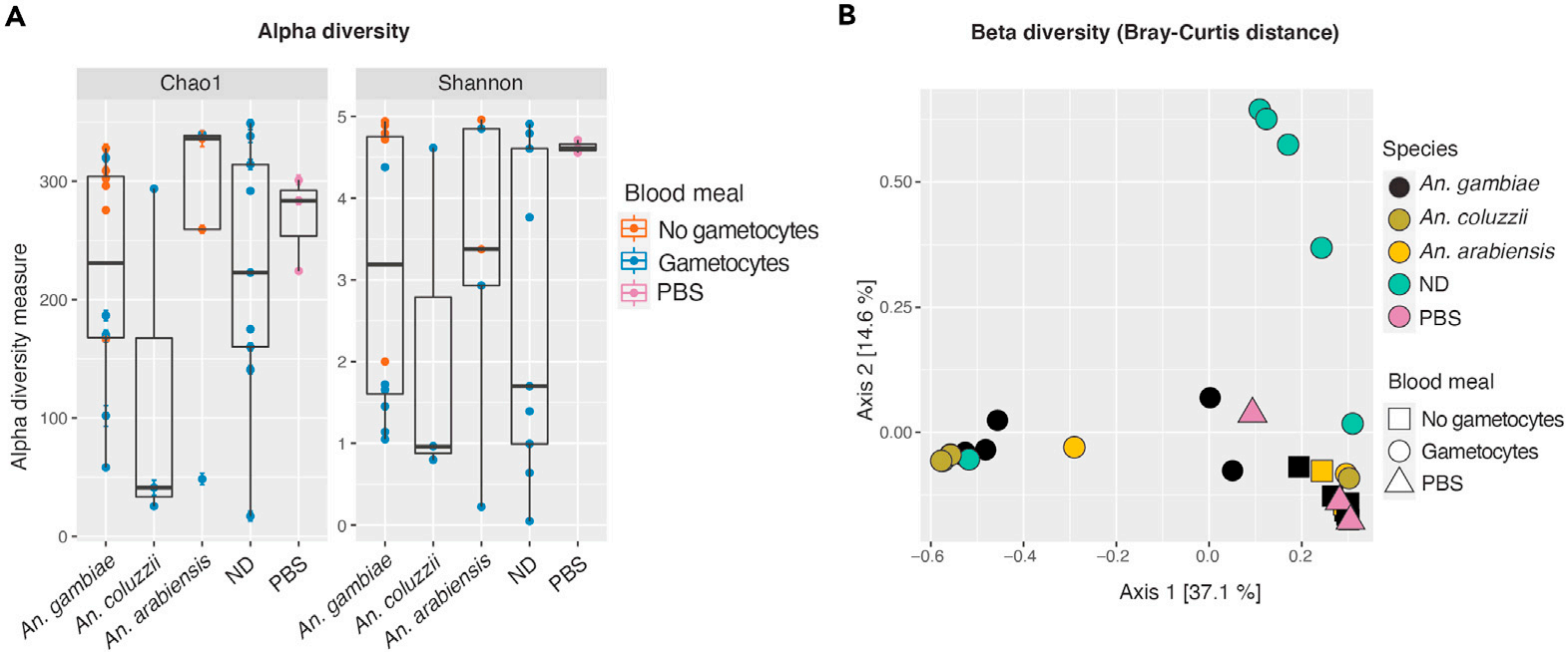
Mosquito species does not significantly influence microbiota composition (A) Alpha diversity of the bacterial gut microbiota of mosquitoes from different *An. gambiae s.l.* species fed with mock-treated blood measured with Chao and Shannon indexes. Single points correspond to individual mosquito indexes and are colored based on whether mosquitoes were fed on *Plasmodium*-infected (blue) or -uninfected (orange) blood; pink indicates PBS negative controls. Black horizontal lines indicate the median, boxplot height indicates the upper and lower quartiles, and whiskers indicate the maximum and minimum values. Error bars in Chao1 plots indicate standard errors. Wilcoxon/Holm, Chao1 index: p = 1.0 for all comparisons; Shannon index: p = 1.0 for all comparisons. ND: nondetermined *Anopheles* species. (B) Beta diversity of the gut bacterial microbiota of mosquitoes from different *An. gambiae s.l.* species represented by a Principal Coordinates Analysis plot of Bray-Curtis dissimilarity. Each point represents a single mosquito. Samples deriving from different mosquito species are highlighted with different colors (yellow: *An. arabiensis*; olive green: *An. coluzzii*; black: *An. gambiae*; green: nondetermined species; pink: PBS negative control). The shape of each point indicates whether mosquitoes were fed on *Plasmodium*-infected (circles) or uninfected (squares) blood (PERMANOVA, F = 1.3, p = 0.14). More data on statistics can be found in Table S4.

We also assessed if the alpha diversity was significantly affected by the mosquito species. Again, this analysis was performed on samples that were not treated with antibiotics. Alpha diversity calculated with both Chao and Shannon indexes was not affected by the mosquito species (Figure 2A, Wilcoxon/Holm, Chao index: p = 1.0 for all comparisons; Shannon index: p = 1.0 for all comparisons). Similarly, Bray-Curtis dissimilarity between samples was not clearly affected by the host species (Figure 2B, PERMANOVA, F = 1.3, p = 0.14).

Finally, we tested whether any interaction between the describing variables (presence of gametocytes in the blood, replicate, time post blood feeding, mosquito species) significantly affected alpha or beta diversities in mock-treated mosquitoes. None of the tested interactions was statistically significant (Table S4).

### Amoxicillin treatment significantly impacts mosquito microbiota composition

**Figure 3:**
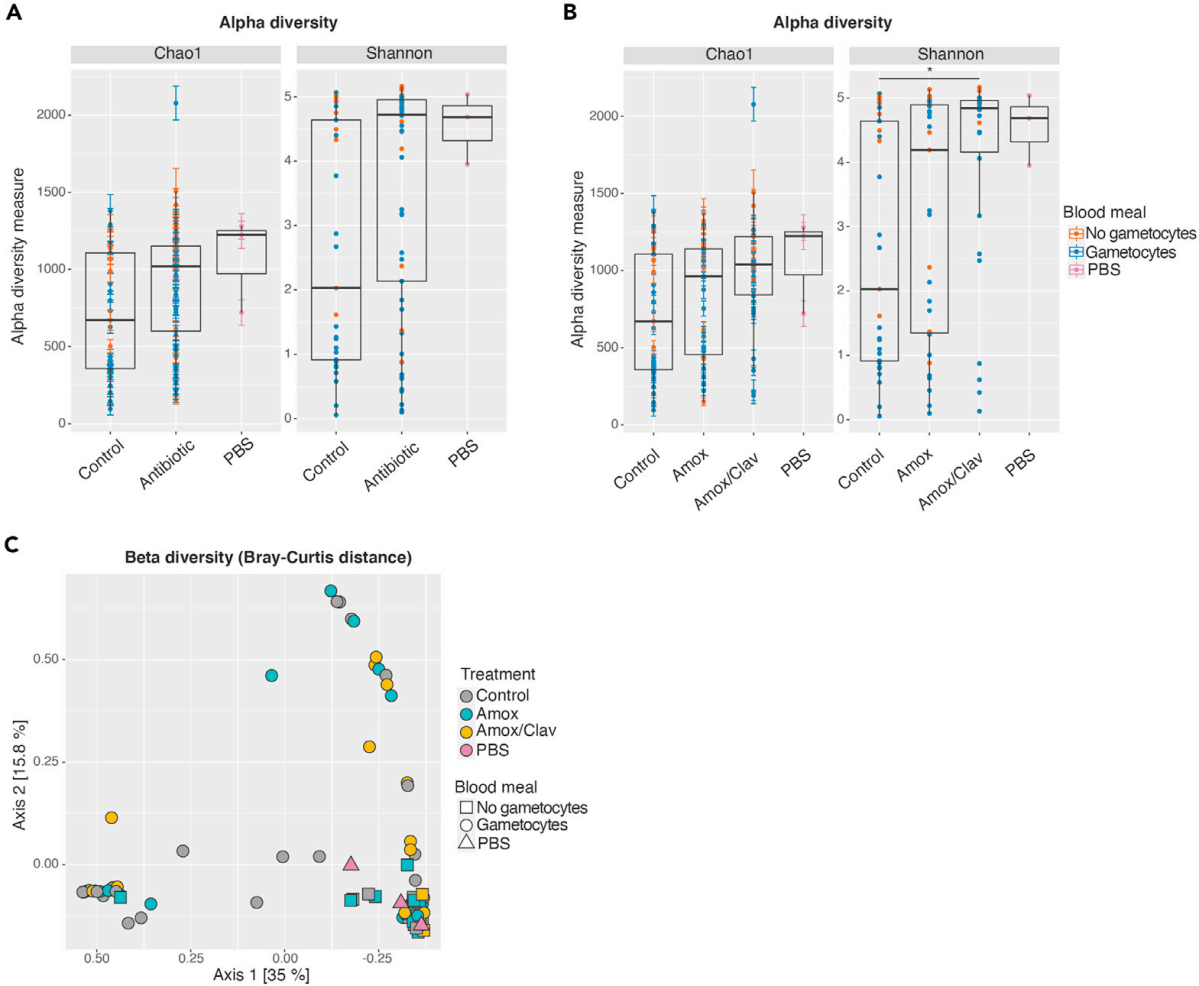
Amoxicillin treatment significantly impacts mosquito microbiota composition (A and B) Alpha diversity of gut bacterial microbiota of *An. gambiae s.l.* mosquitoes fed on blood supplemented with antibiotics or mock-treated, measured with Chao and Shannon indexes. Single points correspond to individual mosquito indexes. Color code corresponds to the presence of *P. falciparum* gametocytes in the blood (orange: uninfected; blue: infected; pink: extraction negative controls). Black horizontal lines indicate the median, boxplot height indicates the upper and lower quartiles, and whiskers indicate the maximum and minimum values. Error bars in Chao1 plots indicate standard errors. In A, mosquitoes fed on blood supplemented with antibiotics are shown together, whereas in B mosquitoes fed on different amoxicillin preparations are shown separately. Wilcoxon/Holm, Chao index, control versus antibiotic p = 0.36; Shannon index, control versus antibiotic p = 0.085 (A); Chao index, control versus Amox p = 1.0, control versus Amox/Clav p = 0.46, Amox versus Amox/Clav p = 1.0; Shannon index, control versus Amox p = 1.0, control versus Amox/Clav p = 0.041, Amox versus Amox/Clav p = 0.35 (B). (C) Beta diversity of gut bacterial microbiota of *An. gambiae s.l.* mosquitoes represented by a Principal Coordinates Analysis plot of Bray-Curtis dissimilarity. Each point represents the microbial composition of a single mosquito. Mosquitoes fed on blood supplemented with different antibiotics are highlighted with different colors (gray: mock-treated blood; blue: Amox; yellow: Amox/Clav; pink: extraction negative controls). The shape of each point indicates the presence of *P. falciparum* gametocytes in the blood (squares: uninfected; circles: infected; triangles: extraction negative controls, PERMANOVA control versus Amox versus Amox/Clav, F = 1.47, p = 0.048; control versus antibiotic, F = 1.63, p = 0.046). Survival analysis upon antibiotic exposure is reported in Figure S3. More data on statistics can be found in Table S4.

We then investigated the effect of exposure to amoxicillin at a high concentration in the blood on the mosquito microbiota. We detected a higher alpha diversity in antibiotic-treated mosquitoes, although this increase in species richness did not reach significance (Figure 3A, Wilcoxon/Holm, Chao index, control versus antibiotic p = 0.36; Shannon index, control versus antibiotic p = 0.085). The alpha diversity indexes in antibiotic-treated mosquitoes were close to those of PBS controls, so we hypothesize that a lower total bacterial load in these samples may have led to a higher detection of rare gut bacteria and/or of environment contaminants.

When specifically investigating the effect of each antibiotic treatment, we found that the combination of amoxicillin and clavulanic acid led to a significant increase in the Shannon index, whereas the impact of amoxicillin alone was not significant (Figure 3B, Wilcoxon/Holm, Shannon index, control versus Amox p = 1.0, control versus Amox/Clav p = 0.041, Amox versus Amox/Clav p = 0.35). The antibiotic treatment also affected beta diversity, whether both amoxicillin treatments are taken separately or together (Figure 3C, PERMANOVA control versus Amox versus Amox/Clav, F = 1.47, p = 0.048; control versus antibiotic, F = 1.63, p = 0.046). In addition, we tested whether an interaction between the antibiotic treatment and any of the other describing variables was significantly affecting the microbial alpha diversity. We found that the interaction between antibiotic treatment and the time of sampling had a significant influence on Shannon index (two-way ANOVA: treatment p = 0.016, time p = 0.60, treatment x time p = 0.018), suggesting that the effects of antibiotics on microbiota composition might be transient. In line with this hypothesis, the interaction between antibiotic treatment and time of sampling on beta diversity was marginally significant (PERMANOVA treatment x time: p = 0.067). All other interactions among variables were not significant on alpha or beta diversity (Table S4).

To assess the effect of antibiotic exposure on mosquito survival, a sub-group of mosquitoes was monitored daily for each replicate (Figure S3). We did not detect any significant impact of antibiotics on mosquito lifespan (log rank Gehan-Wilcoxon test: p = 0.18) although mosquitoes treated with both amoxicillin and clavulanic acid showed a four-day extension in median survival time compared with mock-treated mosquitoes.

### Factors influencing the relative contribution of specific genera

**Figure 4:**
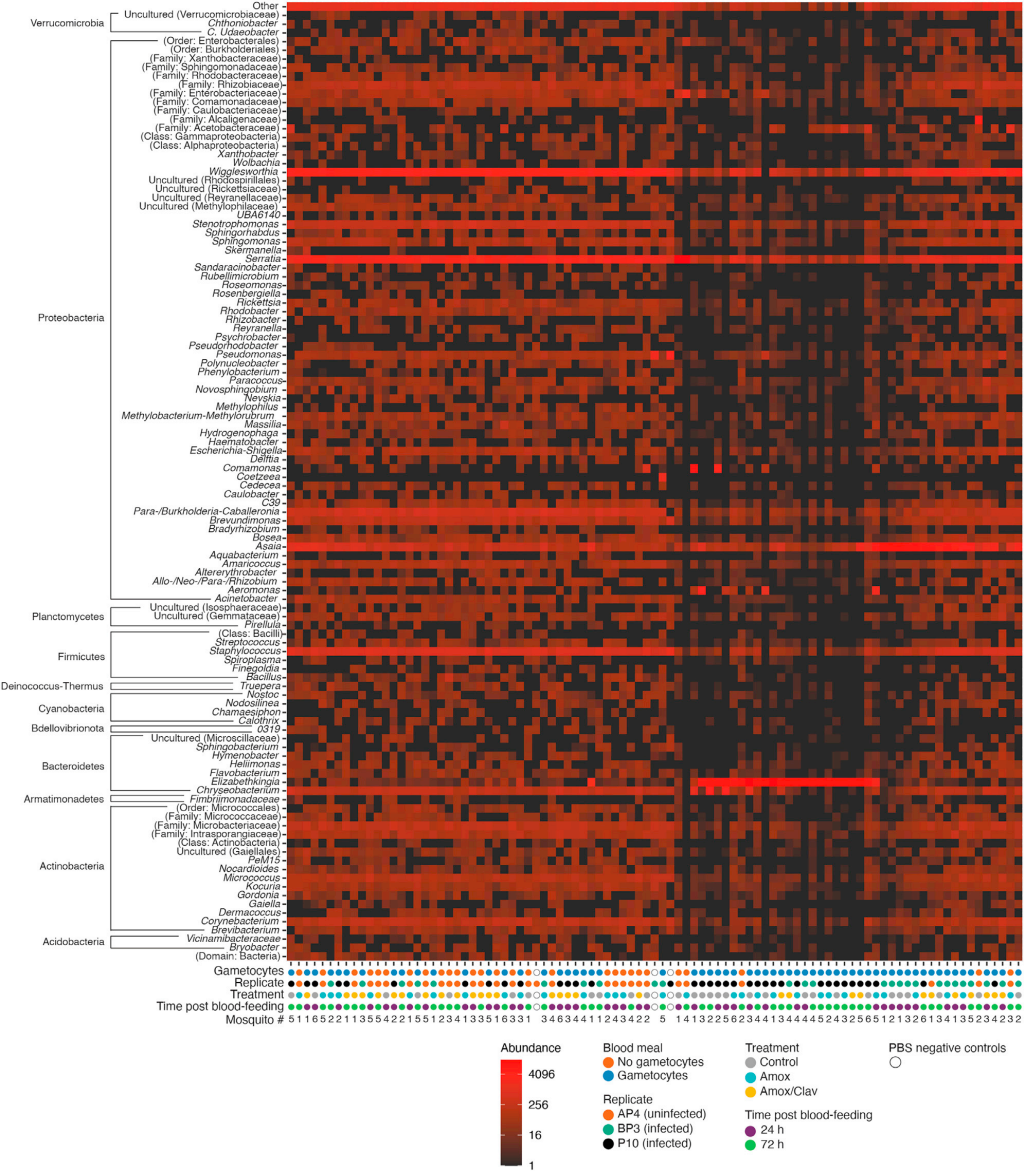
Relative abundance of bacterial genera in *An. gambiae s.l*. mosquitoes Heatmap based on the Bray-Curtis dissimilarity showing the abundance of the detected bacterial genera in the analyzed samples. If the annotation was not available at the genus level, the lowest possible taxonomical level was selected. Below the heatmap, colored dots indicate whether mosquitoes were fed with blood containing *P. falciparum* gametocytes (orange: uninfected, blue: infected), the replicates, named after the corresponding blood donor's code (orange: AP4, green: BP3, black: P10), the antibiotic treatment (gray: control, light blue: Amox, yellow: Amox/Clav), and the sampling time point (purple: 24 h post blood feeding, green: 72 h post blood feeding). White dots indicate extraction negative controls (PBS). See also Figure S4 and Table S3.

The alpha and beta diversity analyses indicated that the main factors influencing the gut microbiota composition were the replicate and, to a lesser extent, antibiotic treatment. Whether the impact of replicates was due to an initial difference in the mosquito microbiota composition, the blood source itself, or the presence of *P. falciparum* gametocytes still has to be assessed. To get some first hints about this, we explored whether the relative abundance of some bacterial genera significantly correlated with these factors. We observed that several genera, notably *Elizabethkingia* (20.1% ± 3.7, average ±SEM), *Wigglesworthia* (17.4% ± 1.2), *Asaia* (11.1% ± 2.3), and *Serratia* (13.0% ± 1.3), were particularly abundant in a number of samples (Figures 4 and S4). To test whether variations in the abundance of each genus are significantly correlated to one or more experimental factors, we applied the DESeq2 algorithm. As the antibiotic treatment and time of sampling seemed to have synergistic effects on microbiota composition, all analyses were performed separately on samples collected at 24 and 72 h after blood feeding.

As a preliminary, we checked whether some bacteria were enriched in negative controls, likely representing a contaminant that was not identified as major during the polishing analysis. We did not identify any genus significantly associated to negative controls, whereas several genera were more abundant in midgut samples (Wald test, PBS versus samples: *Comamonas* log_2_ fold-change (FC) +20.8, *p*adj < 0.001; *Aeromonas* FC +25.1, *p*adj < 0.001; *Elizabethkingia* FC +13.3, *p*adj < 0.001; *Heliimonas* FC +8.4, *p*adj < 0.001; *Cedecea* FC +8.1, *p*adj = 0.0017; *Asaia* FC +4.6, *p*adj = 0.0066).

**Figure 5:**
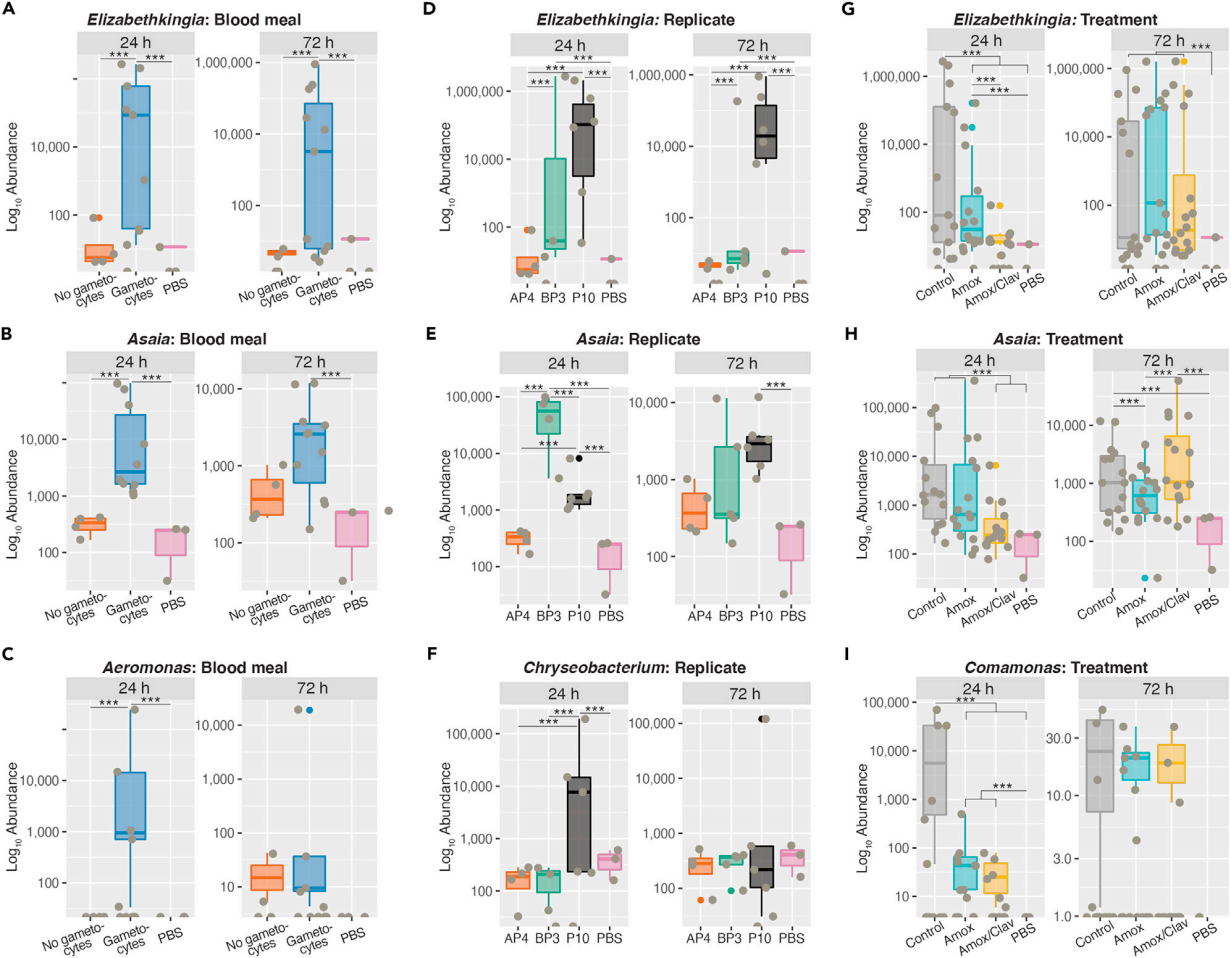
Factors influencing the relative contribution of specific genera (A–C) Relative abundance of *Elizabethkingia* (A), *Asaia* (B), and *Aeromonas* (C) in mosquitoes fed with gametocyte-infected (blue) and uninfected blood (orange) 24 h and 72 h after blood feeding and in PBS samples (pink). Wald test infected versus uninfected: *Elizabethkingia* 24 h and 72 h *p*adj < 0.001; *Asaia* 24 h *p*adj < 0.001; *Aeromonas* 24 h *p*adj < 0.001. See figure for comparisons among samples and PBS controls (∗∗∗: *p*adj < 0.001). All other comparisons: NS. (D–F) Relative abundance of *Elizabethkingia* (D), *Asaia* (E), and *Chryseobacterium* (F) in mosquitoes of different replicates (orange: AP4 [uninfected]; green: BP3 [infected]; black: P10 [infected]; pink: PBS samples) 24 h and 72 h after blood feeding. *Elizabethkingia* Wald test: BP3 versus AP4 24 h and 72 h *p*adj < 0.001; P10 versus AP4 24 h and 72 h *p*adj < 0.001. *Asaia* Wald test: BP3 versus AP4 24 h *p*adj < 0.001; P10 versus AP4 *p*adj < 0.001; BP3 versus P10 24 h *p*adj < 0.001. *Chryseobacterium* Wald test: P10 versus AP4 24 h *p*adj < 0.001; P10 versus BP3 24 h *p*adj < 0.001. See figure for comparisons among samples and PBS controls (∗∗∗: *p*adj < 0.001). All other comparisons: NS. (G–I) Relative abundance of *Elizabethkingia* (G), *Asaia* (H), and *Comamonas* (I) in mosquitoes fed with blood supplemented or not with antibiotics (gray: mock-treated control; light blue: Amox; yellow: Amox/Clav; pink: PBS samples) 24 h and 72 h after blood feeding. *Elizabethkingia* Wald test 24 h: ctrl versus Amox/Clav: *p*adj < 0.001; ctrl versus Amox: *p*adj < 0.001. *Asaia*Wald test 24 h: ctrl versus Amox/clav: *p*adj < 0.001; 72 h: Amox versus Amox/Clav *p*adj < 0.001; *Comamonas* Wald test 24 h: ctrl versus Amox: *p*adj < 0.001; ctrl versus Amox/Clav: *p*adj < 0.001. (A–F) only show data from nonantibiotic-treated mosquitoes. See figure for comparisons among samples and PBS controls (∗∗∗: *p*adj < 0.001). All other comparisons: NS. The bold horizontal line indicates the median, boxplot height indicates the upper and lower quartiles, whiskers indicate the maximum and minimum values. More data on statistics can be found in Table S4.

Then, we investigated the effects of *P. falciparum* gametocytes in the blood meal and of the replicate. To remove potentially confounding factors, the analysis was performed separately on mosquitoes collected 24 h and 72 h after blood feeding and exclusively on mosquitoes fed on mock-treated blood. We found that the genera *Elizabethkingia*, *Asaia*, and *Aeromonas* were significantly enriched in mosquitoes fed on *P. falciparum-*containing blood (Figures 5A–5C, Wald test gametocytes versus no gametocytes: *Elizabethkingia* 24 h FC +14.6, *p*adj < 0.001; 72 h FC +15.9, *p*adj < 0.001; *Asaia* 24 h FC +6.2, *p*adj < 0.001; 72 h NS; *Aeromonas* 24 h FC +32.0, *p*adj < 0.001; 72 h NS). *Elizabethkingia* was more abundant in both groups of mosquitoes fed on blood containing gametocytes (Figure 5D, Wald test: BP3 versus AP4 24 h FC +14.7, *p*adj < 0.001, 72 h FC +13.7, *p*adj < 0.001; P10 versus AP4 24 h FC +4.6, *p*adj < 0.001, 72 h FC +16.6, *p*adj < 0.001). Similarly, the high *Asaia* levels 24 h after blood feeding were explained by the abundance of this genus in mosquitoes fed on blood harboring gametocytes (Figure 5E, Wald test: BP3 versus AP4 24 h, FC +7.4, *p*adj < 0.001; P10 versus AP4 24 h + 3.1, *p*adj = 0.008; BP3 versus P10 24 h FC +4.4, *p*adj < 0.001), whereas this was not observed at 72 h post-blood feeding (Figure 5E). Moreover, *Chryseobacterium* was specifically enriched in mosquitoes of replicate P10 at 24 h but not at 72 h post-blood feeding (Figure 5F, Wald test: P10 versus AP4 24 h FC +7.7, *p*adj < 0.001; P10 versus BP3 24 h FC +8.0, *p*adj < 0.001; 72 h NS). These results confirm the importance of the experimental conditions—whether the date of the experiment, site of mosquito collection, blood donor, and/or donor infection status—on the mosquito microbiota composition.

Secondly, we explored the effect of antibiotic treatment on microbiota composition. At 24 h post-blood feeding, the relative abundances of the genera *Elizabethkingia*, *Asaia*, and *Comamonas* decreased significantly in mosquitoes fed on blood supplemented with Amox and/or Amox/Clav (Figure 5G, *Elizabethkingia* Wald test: ctrl versus Amox FC +7.1, padj < 0.001; ctrl versus Amox/Clav FC +14.2, *p*adj < 0.001; Amox versus Amox/Clav: + 7.1, *p*adj < 0.001; Figure 5H, *Asaia* Wald test: ctrl versus Amox NS; ctrl versus Amox/Clav FC +3.3, *p*adj < 0.001; Amox versus Amox/Clav FC +3.3, *p*adj < 0.001; Figure 5I, *Comamonas* Wald test: ctrl versus Amox FC +7.7, *p*adj < 0.001; ctrl versus Amox/Clav FC +8.8, *p*adj < 0.001; Amox versus Amox/Clav NS). The observed perturbation caused by antibiotic treatment was no longer clear 72 h after blood feeding, as *Asaia* showed an opposite trend compared with the earlier time point (Wald test: ctrl versus Amox NS; ctrl versus Amox/Clav NS; Amox versus Amox/Clav FC -3.2, *p*adj > 0.001), and the other bacterial genera were not reproducibly affected at this time (Figures 5G–5I). This indicate that antibiotic treatment does not have any long-lasting negative impact on these bacteria after the end of the digestion of the treated blood meal.

## Discussion

In this study, we describe variations in the microbiota composition of *An. gambiae s.l.* mosquitoes reared in semi-field conditions, fed on blood containing or not containing *P. falciparum* gametocytes, with or without amoxicillin.

In microbiota studies, caution should be taken to avoid the introduction of contaminant microbes in the samples (Dada et al., 2021). However, the extraction and amplification of microbial genetic material present in the environment and in laboratory reagents is a common problem, especially when dealing with low microbial biomass samples such as individual mosquito midguts (Eisenhofer et al., 2019; Karstens et al., 2019). To monitor the presence of contaminating microbes, we included negative controls that were manipulated and sequenced together with midgut samples, as conventionally performed. Previous works aiming at characterizing the mosquito microbiota composition used different approaches to deal with bacterial contaminants: some studies removed from the analysis all the ASVs identified in negative controls (Dickson et al., 2018); some others used abundance-based criteria to identify contaminants and remove them (David et al., 2016; Díaz et al., 2021; Dickson et al., 2017; Minard et al., 2015) or just acknowledge them as contaminants (Caragata et al., 2021; Gendrin et al., 2015, 2016); other studies used R packages specifically implemented to identify contaminant ASVs (Frankel-Bricker et al., 2020; Frankel-Bricker and Frankel, 2021). However, a consensus on the best method to deal with contaminants is still missing; notably data from negative samples are scarcely mentioned in main manuscripts, and they can only be found in supplemental files and/or in databases. We decided to use a combinatory approach based on (1) the identification of major contaminant ASVs according to their prevalence in all samples and their abundance in negative controls compared with true samples and on (2) a thorough reporting of remaining negative sample data throughout the manuscript.

Considering the contaminant identification step, this process led to the elimination of six major contaminant ASVs, which represented on average the 23% of the bacterial diversity. Because raw read numbers of our dataset were similar across controls and samples, the use of raw read counts and relative abundance led to similar results. However, this step should be evaluated for each dataset. Contaminant ASVs belonged to the main bacterial genera identified in midgut samples (*Wigglesworthia* and *Chryseobacterium*). Indeed, bacterial sequences corresponding to *Chryseobacterium*, *Staphylococcus*, or *Rhizobium* have been previously identified as contaminants of laboratory reagents (Glassing et al., 2016; Salter et al., 2014), whereas *Wigglesworthia* is not a typical contaminant. As this bacterium is a tsetse fly symbiont not previously reported in the mosquito microbiota, we even wondered if the other *Wigglesworthia* reads may just result from contamination. However, no research on tsetse flies is performed in the laboratory where samples were processed. The sequencing flow cell contained our samples only, and the sequencer is cleaned at the beginning and at the end of each run, so we do not see where such a high contamination would come from. Hence, it is possible that the corresponding ASVs are actually cross-contaminants from samples. Further work will be needed to further assess if *Wigglesworthia* (or a taxonomically close bacterium) is a member of the mosquito microbiota. Overall, the suitability of our method is supported by the clearer discrimination between true samples and negative controls after the removal of these ASVs and by the absence of specific bacterial genera enriched in negative controls. Alternatively, considering as contaminants all the ASVs detected in PBS controls would have led us to remove an *Elizabethkingia* ASV present in 85% of the samples with an average contribution of 19% in samples (0–99%) and of 0.01% in controls, underlining the importance to tune the contaminant sorting parameters. However, some midgut samples showed a microbial composition close to negative controls even after the removal of these major contaminants. Because it is impossible to determine if DNA extraction failed in these samples, if they carried a microbial load that was under the level of detection, or if a cross-contamination happened, we argue for the inclusion of negative controls in all downstream analyses, similar to what is shown in all types of experimental data.

Considering this control-reporting step, monitoring data from negative results besides data from samples allows to critically evaluate results and avoid misinterpretations. For instance, comparing alpha and beta diversities of true samples and negative controls led us to consider potential extraction/amplification problems in AP4 samples rather than to draw any strong conclusion on the apparent lower bacterial diversity of mosquitoes fed on gametocyte-carrying blood. Recently, the need to improve methods of analysis and report in mosquito microbiota research has been raised (Dada et al., 2021). We believe that reporting of data from negative control would improve interpretation of data in our field and in any study on low microbial-biomass samples.

We tested how amoxicillin and amoxicillin combined to clavulanic acid in the blood meal affect mosquito microbiota composition because these antibiotics are extensively used to treat febrile symptoms in Sub-Saharan Africa (Blaise Savadogo et al., 2014). Our data reveal that *An. gambiae s.l.* gut microbiota composition is affected upon exposure to these antibiotics via the blood meal, with an overall impact on alpha and beta diversity and a more specific reduction in *Elizabethkingia*, *Asaia*, and *Comamonas* abundance. Only a mild nonsignificant increase in survival was observed in mosquitoes treated with amoxicillin and clavulanic acid. Previous studies described different impacts of antibiotics in the blood meal on mosquito fitness and microbiota composition. Exposure to penicillin-streptomycin at therapeutic concentration decreased microbiota alpha diversity and increased mosquito survival and reproduction success in *An. coluzzii* (Gendrin et al., 2015). Doxycycline had a nonsignificant positive effect on mosquito lifespan, whereas azithromycin decreased mosquito lifespan and co-trimoxazole did not affect mosquito survival (Gendrin et al., 2016). Caution should be taken when comparing different studies investigating the effect of antibiotics on the mosquito microbiota, as the observed effects are affected by the initial composition of gut bacterial communities and by antibiotic concentrations. Moreover, samples of these former two studies resulted from pooled mosquitoes, hence the issue of contamination was less serious than in the current study. However, taken together our comparison between two amoxicillin preparations confirm previous observation that antibiotics have a drug-specific impact on the bacterial composition of the mosquito microbiota and on mosquito lifespan.

The reduction in *Elizabethkingia*, *Asaia*, and *Comamonas* upon amoxicillin treatment was observed 24 h but not 72 h after the blood meal. This plasticity of the mosquito microbiota has already been reported (Gendrin et al., 2015) and is likely due to the expulsion of most gut bacteria with the fecal pellet at the end of digestion (Rodgers et al., 2017). A positive impact on *Asaia* is however observed at 72 h, similar to the higher bacterial load previously reported after a second blood-meal after treatment with doxycycline (Gendrin et al., 2016), suggesting that long-lasting dysbiosis cannot be ruled out or that the lower induction in gut immunity may lead to a higher bacterial proliferation in later stages. These results together suggest that time points corresponding to the microbiota acme, around 24 h after blood meal, are particularly appropriate to detect the effects of antibiotics on mosquito microbiota composition and that milder long-term effects of antibiotics can sometimes be observed after pellet expulsion and/or after next blood meal.

Our data suggest that mosquitoes fed on amoxicillin-/clavulanic-acid-containing blood show a significant perturbation of their microbiota, which may affect vectorial capacity (Dong et al., 2009; Gendrin et al., 2015; Martínez-de la Puente et al., 2021). We used semi-field mosquitoes in order to assess the impact of antibiotics with a relevant model. Further studies should investigate the effect of amoxicillin on *Plasmodium* infection and test whether similar effects on the microbiota are observed when using amoxicillin at therapeutic doses. As antibiotic concentration in the plasma does not directly reflect the concentration added to the blood and as antibiotic-concentration assays were not possible, we used as a proof-of-concept the maximum antibiotic concentration on which mosquitoes would efficiently blood feed, to assess whether amoxicillin can induce a microbiota perturbation in mosquitoes. Indeed, the estimated human blood plasma concentration for amoxicillin is 0.5–15 mg.L^−1^ after amoxicillin ingestion (Schulz et al., 2020), whereas the amoxicillin concentration in the whole blood used in this study, 860 mg.L^−1^, is much higher. Although our data suggest a significant shift in the mosquito microbiota composition after the ingestion of amoxicillin-supplemented blood, further experiments are required to assess whether these effects are still observed at antibiotic therapeutic doses.

Our samples were characterized by two dominant phyla (Proteobacteria—62% and Bacteroidota—23%) and four different genera (*Elizabethkingia*, *Wigglesworthia*, *Asaia*, and *Serratia*). Using culture-dependent methods, we previously identified several microbiota members of *An. gambiae s.l.* mosquitoes kept in semi-field conditions and noted that *Asaia* and *Serratia*, which belong to Proteobacteria, were predominant (Yerbanga et al., 2017). *Elizabethkingia* has been reported by several studies as part of the microbiota of field-collected mosquitoes. As mentioned earlier, *Wigglesworthia* is not typically found in mosquitoes, and further investigation of its presence of the mosquito microbiota would be of interest. Other studies on the microbiota composition of field mosquitoes in Burkina Faso identified Proteobacteria as the dominant phylum, with *Enterobacter*, *Aeromonas*, and *Pseudomonas* or *Acinetobacter* and *Pseudomonas* being the most prevalent genera (Straub et al., 2020; Zoure et al., 2020). These variations in terms of genus composition might be related to differences in experimental settings such as the source of mosquitoes analyzed, as ours were collected in the field as larvae and reared in the insectary as adults, whereas other studies, which did not require any experimentation prior to sampling, were based on field mosquitoes. Moreover, we worked on individual midguts, whereas previous work has been performed on pools of midguts or headless carcasses.

We tested if any correlation can be identified between variable factors among our samples and microbiota composition of *An. gambiae s.l.* mosquitoes. The mosquito species was not associated to any specific microbiota composition, and mosquitoes belonging to *An. arabiensis, An. gambiae*, or *An. coluzzii* species and reared in the same larval breeding site carried similar gut microbes. This observation is in line with several independent studies that reported no correlation between mosquito species and microbiota composition and that reported that sampling location and breeding site are more relevant in determining the structure of microbial gut communities (Coon et al., 2016; Gimonneau et al., 2014; Muturi et al., 2017; Straub et al., 2020).

In our conditions, the experimental replicate and, to a lesser extent, the antibiotic treatment significantly affected the microbial gut community structure. Considering untreated samples, mosquitoes from different replicates, i.e. collected in distinct sites at different time points and fed on different blood sources, showed specific microbiota compositions, with *Elizabethkingia, Asaia*, and *Chryseobacterium* being the most variable genera. It is however difficult to determine whether these differences are due to the microbial composition of their original breeding sites (and consequently of resulting adults), characteristics of the blood fed to mosquitoes (presence of *Plasmodium* gametocytes or other blood properties), or other random effects. As previously reported, sampling location, seasonality, and characteristics of the breeding sites are major factors influencing the mosquito microbiota composition (Coon et al., 2016; Gimonneau et al., 2014; Muturi et al., 2017; Straub et al., 2020). In addition, specific mosquito microbiota compositions correlated with *Plasmodium* infection, such as the high abundance of the Enterobacteriaceae family (Boissière et al., 2012). Although *Plasmodium*-gametocyte ingestion did not lead to any successful parasite development in our experiments, our data indicate a positive correlation between the Weeksellaceae and Acetobacteraceae families and the presence of *Plasmodium* gametocytes in the blood meal. It would be interesting to explore whether this co-occurrence is reproducible in similar experimental conditions, for instance using mosquitoes with a simplified and known microbiota composition (i.e. gnotobiotic mosquitoes). Such correlations may relate to specific bacterium-parasite metabolic interactions or to indirect effects of parasites on blood and/or mosquito tissues. Finally, variations in mosquito microbiota composition may be influenced by specific characteristics of the ingested blood. Feeding on different blood donors impacts *An. gambiae* longevity (Sangare et al., 2013), suggesting a variability in blood components or metabolites that are biologically significant and hence may also impact the microbial gut community structure.

In conclusion, we analyzed the midgut microbiota of 90 mosquitoes collected as larvae in Burkina Faso and fed as adults with blood in the presence or absence of *Plasmodium*-gametocytes and of amoxicillin treatments. Working on very small DNA extracts led us to rethink the way we treat negative controls, and we propose that identifying some contaminants should not preclude the explicit presentation of negative controls. Our results indicate that amoxicillin and amoxicillin/clavulanic acid added to the blood at a high concentration strongly reduced the major microbiota members *Elizabethkingia*, *Asaia*, and *Comamonas*, which should be further investigated at therapeutic concentrations. We observed significant variations between replicates, which are consistent with an impact of gametocyte carriage in the ingested blood on the microbiota, but this would require further validation. We also confirmed previous observation that the microbiota composition is not shaped by mosquito species in the *An. gambiae* s. l. complex.

### Limitations of the study

Although we used a methodology closely related to field conditions, the adult microbiota may be influenced by the laboratory environment where mosquitoes were kept after emergence. Moreover, further work is still required to assess whether the effect of amoxicillin is confirmed with lower drug concentrations. Finally, the correlation between gametocyte carriage and microbiota composition should not be overinterpreted. So far it is a correlation, and further work is needed to assess whether *Plasmodium* may affect microbiota composition.

## STAR★Methods

### Key resources table

**Table:** 

REAGENT or RESOURCE	SOURCE	IDENTIFIER
**Biological samples**		

Vein blood	Informed volunteers	N/A

**Chemicals, peptides, and recombinant proteins**		

Baby fish-food (Tetramin®)	Tetra GmbH	Cat#: T509339 FR, EP 0848592
Glucose	Biosolve	Cat#: 07142391, Lot: 961061
Giemsa	Quimica Clinica Aplicada	Cat#: 990939, Lot: 180650
Lithium-heparin tube	Vacutest Kima	Cat#: 12010, Lot: J0527
Amoxicillin	Humanwell	Cat#: N/A, Lot: 180415
Amoxicillin and clavulanic acid	ZENTIVA, Sanofi	BN: N180116
Saline solution	Miniversol	Cat#: 69064589
PBS	ThermoFisher	Cat#: J61196, Lot: N20D502
Mercurochrome	Sigma-Aldrich	Cat#: M7011-25G, Lot: 028K0724
Taq DNA polymerase	Qiagen	Mat No: 1005476, Lot: 148028435
KAPA HiFi HotStart ReadyMix	Roche	Cat#:07958935001, N/A

**Critical commercial assays**		

Rapid diagnostic tests (SD Bioline Malaria HRP2-II Pf and pLDH Pan)	Abbott	Cat#: 05FK60, Lot: 05EDF030A
DNeasy Blood and Tissue kit	Qiagen	Cat#: 69506, Lot: 163043355

**Deposited data**		

Raw sequencing data	This paper	PRJEB45342 (ENA)
R code for statistical analyses	This paper	https://doi.org/10.5281/zenodo.5109331
16S SILVA database	(Quast et al., 2013)	https://www.arb-silva.de/

**Experimental models: Organisms/strains**		

*Anopheles gambiae s.l.*	Field (Burkina Faso)	NCBI:txid44542

**Oligonucleotides**		

S200X 6.1F (5’-TCG-CCT-TAG-ACC-TTG-CGT-TA-3’)	(Santolamazza et al., 2008)	N/A
S200X 6.1R (5’-CGC-TTC-AAG-AAT-TCG-AGA-TAC-3’)	(Santolamazza et al., 2008)	N/A
343F (5’-TAC-GGG-AGG-CAG-CAG-3’)	(Herlemann et al., 2011)	N/A
806R (5’-GGA-CTA-CCA-GGG-TAT-CTA-AT-3’)	(Herlemann et al., 2011)	N/A

**Software and algorithms**		

FastQC	(Andrews, 2019)	https://www.bioinformatics.babraham.ac.uk/projects/fastqc/
Trimmomatic	(Bolger et al., 2014)	http://www.usadellab.org/cms/?page=trimmomatic
QIIME2	(Callahan et al., 2016)	https://qiime2.org/
R Project for Statistical Computing	The R project	https://www.r-project.org/ RRID:SCR_001905
Decontam package	(Davis et al., 2018)	https://www.bioconductor.org/packages/release/bioc/html/decontam.html
Phyloseq package	(McMurdie and Holmes, 2013)	https://joey711.github.io/phyloseq/
Survival package	Therneau et al., 2000	https://github.com/therneau/survival

### Resource availability

#### Lead contact

Further information and requests for resources and reagents should be directed to and will be fulfilled by the lead contact, Mathilde Gendrin (mathilde.gendrin@pasteur.fr)

#### Materials availability

This study did not generate new unique reagents.

### Experimental model and subject details

#### Human subjects

Five-to-12-year-old asymptomatic children from Matroukou and Dandé were screened for the presence of *P. falciparum* gametocytes in the blood. Two gametocyte carriers (BP3 and P10) and one uninfected donor (AP4) were selected. The sex and age of the three donors were an 11-year-old male (BP3), a 5-year-old female (P10) and 10-to-12 –year-old male (AP4). All procedures in this study including human subjects have been approved by the Institutional Ethics Committee for the Research of Health Sciences (Comité d’Ethique Institutionnel pour la recherche en Sciences de la Santé: CEIRES) under the ethical clearance number 2020-19/MESRSI/CNRST/IRSS/CEIRES. Gametocyte carriers were volunteers and their parents had accepted to sign a written informed consent before enrollment.

#### *Anopheles gambiae* sensu lato

Mosquito larvae were sampled in five natural breeding sites in Burkina Faso during three collection campaigns. During each collection, larvae were collected in 5 L containers and transferred to the Institut des Sciences et Techniques (INSTech) in Bobo-Dioulasso. Anopheline larvae (NCBI: tax_id 44542) were morphologically identified while other larvae and predators were discarded. Larvae were reared outdoor in clay jars filled with 500 mL water from their own breeding sites and fed daily with 0.3 mg Baby fish-food (Tetramin®, Tetra GmbH) per larva. Pupae were transferred with their breeding water in sterile plastic cups during three consecutive days and placed in 30 cm x 30 cm x 30 cm cages inside the insectary for emergence. Adult mosquitoes were kept in standard insectary conditions (Temperature: 27 ± 2°C, Humidity: 80 ± 5% RH and light/dark: 12 h) and fed with 5% sterile glucose solution (Biosolve). The next steps were performed on females only, as males do not blood feed. The effect of the presence of gametocytes and antibiotics in the blood on the mosquito microbiota was tested on five-to-six-day-old-female mosquitoes previously starved for 48 h and fed for 1 h on blood collected from human patients. For each replicate (i.e. blood donor and collection campaign), mosquitoes were randomly allocated to the three treatments (mock control, amoxicillin, amoxicillin/clavulanic acid) and 50 mosquitoes per treatment were offered a blood meal. Only blood fed mosquitoes were selected for microbiota and survival analyses. Mosquito species was determined on dissected carcasses via PCR (see Mosquito species identification paragraph). All procedures in this study including mosquito maintenance have been approved by the Institutional Ethics Committee for the Research of Health Sciences (CEIRES) under the ethical clearance number 2020-19/MESRSI/CNRST/IRSS/CEIRES.

### Method details

#### Mosquito collection

Mosquito larvae were sampled in five natural breeding sites in Pè, a peri-urban area of Bobo-Dioulasso, and around villages (Hounde, Kari, Sebedougou, Koumbia) in Burkina Faso (Figure S1) using standard dipping techniques. The collection was performed during the end of the rainy season in September, October and December 2019. Details are shown in Table 1. During each collection campaign, larvae were collected in 5 L containers and transferred to the Institut des Sciences et Techniques (INSTech) in Bobo-Dioulasso. Anopheline larvae were morphologically identified while other larvae and predators were discarded. Larvae were reared outdoor in clay jars filled with 500 mL water from their own breeding sites and fed daily with 0.3 mg Baby fish-food (Tetramin®, Tetra GmbH) per larva. Pupae were transferred with their breeding water in sterile plastic cups during three consecutive days and placed in 30 cm x 30 cm x 30 cm cages inside the insectary for emergence. The remaining larvae were discarded to avoid potential bias related to the modification of microbiota after being kept for too long outside their natural habitat. Adult mosquitoes were kept in standard insectary conditions (Temperature: 27 ± 2°C, Humidity: 80 ± 5% RH and light/dark: 12 h) and fed with 5% sterile glucose solution (Biosolve).

#### Blood collection and experimental infection

Five-to-12-year-old asymptomatic children from Matroukou and Dandé, two villages close to Bobo-Dioulasso, were screened for the presence of *P. falciparum* gametocytes in the blood. Rapid Diagnosis Tests (RDTs, SD Bio Line, Malaria HRP2-II (Pf) and pLDH (Pan), 05EDF040A) were performed on site to select *P. falciparum* carriers. Thick smears were then prepared for people with positive RDTs, transported to the laboratory and stained with Giemsa (Quimica Clinica Aplicada) at 10% and examined under light microscope (1000x) to detect *P. falciparum* gametocytes. At the time of the experiment (generally the next day), new thick smears were performed on peripheral and venous blood of the selected gametocyte-carrier to determine parasitemia. For the experiment with uninfected blood, RDT and thick smears were also performed to ensure the absence of *Plasmodium*.

Three donors were selected to perform the study. They stated orally that they had not received any treatment with antimalarials or antibiotics (whether conventional drugs or herbal medicine) during at least ten days before the experiment. A single gametocyte carrier was used per experiment to avoid donor-dependent variability in infection rate. For each donor, vein blood was collected in a lithium-heparin tube (Vacutest KIMA) and kept at 37°C. The effect of antibiotics on the mosquito microbiome was tested using two forms of amoxicillin-based treatments, one consisting of amoxicillin alone (HUMANWELL, referred to as Amox) and one consisting of a combination of amoxicillin and clavulanic acid (Fleming® 1200 mg; referred to as Amox/Clav). Amoxicillin is a β-lactam antibiotic targeting the peptidoglycan synthesis in bacteria, while clavulanic acid is an inhibitor of β-lactamase enzymes and it is often used in combination to β-lactam antibiotics to hamper antibiotic-resistance in β-lactamase-producing bacteria. For each treatment, 1000 mg of amoxicillin was dissolved in 20 mL of saline solution (NaCl 0.9% w/v, Miniversol) to obtain 50 mg.mL^-1^, and further diluted to 12.5 mg.mL^-1^. 68.5 μL was added to 1 mL on infected blood to have a final concentration of 0.86 mg.mL^-1^ and incubated at 37°C during 1 h. Control blood was supplemented with 68.5 μL of saline solution. This concentration was chosen as the maximum concentration in the blood on which mosquitoes would still blood feed. It is 100-1000 times the peak concentration observed in human plasma after amoxicillin ingestion (Schulz et al., 2020).

Five-to-six-day-old-female mosquitoes were starved for 48 h and fed for 1 h on infectious blood through Direct Membrane Feeding Assay and using Parafilm® coated feeders maintained at 37°C. For each infection, 50 mosquitoes per treatment were offered a blood meal. After feeding, fully blood-fed mosquitoes were sorted and kept in standard insectary conditions until dissection. Survival was monitored daily for all mosquitoes until sampling (day 1 or 3 for microbiota analysis and day 8 for *Plasmodium* infection) and on the remaining mosquitoes thereafter.

#### Mosquito dissection and DNA extraction

Dissections were carried out in sterile PBS. To evaluate the effect of the treatment on the composition of the bacterial gut microbiota, midguts of individual mosquitoes were dissected 24 h and 72 h after blood feeding. More precisely, we defined the midgut as the region between the cardia (included) and the Malpighian tubules (excluded). Mosquitoes were cold-anesthetized, kept in 70% ethanol for 5 minutes (to kill surface bacteria and stick them to the cuticle) and rinsed three times in sterile phosphate buffer saline (PBS, ThermoFisher). Guts were dissected on a drop of sterile PBS and stored individually at -20°C in 50 μL sterile PBS. In parallel, carcasses were stored individually at -20°C until processing for species identification. Dissection tools (forceps and slides) were sterilized between each sample using 70% ethanol and rinsed in sterile PBS. DNA extraction was performed inside a microbiological safety cabinet. Genomic DNA from individual guts and carcasses was extracted using the DNeasy Blood and Tissue kit (Qiagen) according to manufacturer’s instructions and eluted in 50 μL sterile water. DNA samples were stored at -20°C until analysis. Controls consisted of 50 μL of the same aliquot of sterile PBS used for individual midgut storage in a sterile microcentrifuge tube. Each replicate included a negative-control tube, treated as the samples throughout the experiments.

To monitor the impact of the treatment on infection, mosquitoes of the September and December replicates were dissected 8 days after the infectious blood meal. For oocyst counts, midguts were dissected and stained with 1% mercurochrome (Sigma-Aldrich) while for sporozoite counts, salivary glands were dissected and observed without any staining. However, these results could not lead to any conclusion as infection was undetectable or low: 0/25 and 0/50 mosquitoes carried any oocysts in September and December, respectively; 3/44 mosquitoes carried some sporozoites in December (6 salivary glands dissections failed). Sporozoite load was not tested in September.

#### Mosquito species identification

*An. gambiae* s.l. species was determined by PCR on DNA samples extracted from mosquito carcasses following the protocol of Santolamazza et al. (Santolamazza et al., 2008). Briefly, amplification was performed using Qiagen Taq DNA polymerase according to the manufacturer’s protocol with the following specific primers: S200X 6.1F: 5’-TCG-CCT-TAG-ACC-TTG-CGT-TA-3’ and S200X 6.1R: 5’-CGC-TTC-AAG-AAT-TCG-AGA-TAC-3’. 2μL of DNA was amplified using the following program: 94°C for 10 min, 35 cycles of 94°C for 30 s, 54°C for 30 s and 72°C for 60 s. Species were identified according to amplicon size (479 bp for *An. coluzzii*, 249 bp for *An. gambiae,* 223 bp for *An. arabiensis*)*.*

#### Library preparation and sequencing

The microbial composition of the mosquito gut microbiota was investigated by sequencing the V3 and V4 regions of the bacterial *16S* rRNA gene. PCRs and sequencing were performed by Polo d’Innovazione Genomica Genetica e Biologia (Siena, Italy). The couple of primers 343F (5’-TAC-GGG-AGG-CAG-CAG-3’) and 806R (5’-GGA-CTA-CCA-GGG-TAT-CTA-AT-3’) (Herlemann et al., 2011) was used to amplify the *16S* gene of 90 individual gut samples and of 3 negative controls (Table S1). Both forward and reverse primers included overhang Illumina adapters (forward overhang: 5’-TCG-TCG-GCA-GCG-TCA-GAT-GTG-TAT-AAG-AGA-CAG-3’; reverse overhang: 5’-GTC-TCG-TGG-GCT-CGG-AGA-TGT-GTA-TAA-GAG-ACA-G-3’).

PCR amplification was performed on 2.5 μL of template DNA, using 1 μM of each primer, 12.5 μl of ready mix (2x KAPA HiFi HotStart ReadyMix) and using the following amplification protocol: 95°C for 3 min; 25 cycles of 95°C for 30 s, 55°C for 30 s, 72°C for 30 s; 72°C for 5 min. Amplicons were purified using AMPure XP beads (Beckman Coulter). Sequencing libraries were created using the Nextera XT Index kit (Illumina) introducing sample-specific indexes with the following amplification protocol: 8 cycles of 95°C for 30 s, 55°C for 30 s, 72°C for 30 s and a final elongation at 72°C for 5 min. After a second purification step using AMPure XP beads, libraries were quantified using a Qubit fluorometer (Thermo Fisher Scientific). The quality of the final libraries was evaluated by capillary electrophoresis using a Fragment Analyzer System (Agilent Technologies). Indexed libraries were normalized and pooled at a final concentration of 4 nM. The pool was sequenced on an Illumina MiSeq with 20% PhiX control using the MiSeq 2x250 paired-end sequencing kit. A total of 8,430,397 sequences was obtained.

#### Sequence processing and microbial diversity analysis

Raw reads were demultiplexed and their quality was inspected using FastQC (Andrews, 2019). Trimmomatic (Bolger et al., 2014) was used to trim low-quality reads (parameters: SLIDINGWINDOW: 4:20 MINLEN: 36 LEADING:20 TRAILING:20). The QIIME2 cutadapt plugin was used to remove primer and adapter sequences and the DADA2 plugin was used to dereplicate reads, identify chimera and merge paired-end reads (Callahan et al., 2016). A total number of 19,320 ASVs were identified in all the samples. In order to assign the taxonomy to each ASV, a classifier was trained through machine learning using the QIIME2 classify-sklearn plugin. The classifier was then applied to the 16S SILVA database (Quast et al., 2013) to select only the V3-V4 region of the *16S* sequence. The amplicon sequences were then matched with the refined database and the taxonomy assigned to each ASV. A further filtering step was performed to remove 2,571 ASVs corresponding to mitochondrial and chloroplast sequences (plugin: taxa filter-table).

To identify major contaminant ASVs, the composition of the 3 negative controls was analyzed. ASVs were considered major contaminants and removed when they were present in more than 80% of samples and when their average abundance in the 3 negative controls was higher than their average abundance in all the samples. Contaminants identified with this method were compared to those identified with the “decontam” package (version 1.6.0, (Davis et al., 2018)). In total, 6 ASVs were removed at this step (Table S2). Negative controls were also included in all the further analyses and figures to consider the other contaminants throughout this analysis.

### Quantification and statistical analysis

#### Statistical analysis

Statistical analyses were performed in R (version 3.6.0). Analyses on microbiota composition were carried out with the “Phyloseq” package (version 1.28.0, (McMurdie and Holmes, 2013). A Wilcoxon rank sum test was used to compare alpha diversity measures and Holm correction was applied for multiple comparisons (referred as Wilcoxon/Holm). A two-way ANOVA was used to investigate the interactions between variables in describing alpha diversity measures. A permutational multivariate analysis of variance (PERMANOVA) was used when comparing beta diversity measures (Bray-Curtis dissimilarity indexes), as well as interactions between variables. Differential abundances on genera were tested with the DESeq2 package included in Phyloseq using a Wald test. The analysis of mosquito survival was performed using the “survival” package (Therneau et al., 2000; version 3.2) and a Gehan-Wilcoxon log-rank test was used to compare survival curves of mock mosquitoes and mosquitoes treated with antibiotics. All statistical details are specified in the results section and figure legends. Detailed results of statistical tests are reported in Table S3. Blinding was not relevant for this study and power analysis was not performed before initiating the study.

## Data Availability

• Datasets supporting this study have been deposited at European Nucleotide Archive and are publicly available as of the date of publication. Accession numbers are listed in the key resources table. • All original code has been deposited at Zenodo and is publicly available as of the date of publication. DOIs are listed in the key resources table. • Any additional information required to reanalyze the data reported in this paper is available from the lead contact upon request.

## Supplemental information

Document S1. Figures S1–S4 and Table S1

Table S2. Description of the negative controls, related to STAR Methods Contaminant ASVs identified with the "decontam" package and the method described in this study. For each ASV the following information is reported: id code, taxonomy, result of the "decontam" analysis, result of our analysis on abundance data, average abundance in samples, maximum value of abundance in samples, minimum value of abundance in samples, average abundance in negative controls, result of our analysis on raw count data, average raw count in samples, maximum value of raw counts in samples, minimum value of raw counts in samples, average raw counts in negative controls, prevalence in samples, and prevalence in negative controls. A thick black line shows the threshold used in our method (prevalence of the ASV in the 80% of samples).

Table S3. Raw read counts and taxonomy of each ASV identified in this study, related to Figure 4

Table S4. Statistics related to Figures 1, 2, 3, 5, and S2 Data of a sheet correspond to a figure.
