# Supplementary material for: Analyzing gut microbiota composition in individual Anopheles mosquitoes after experimental treatment
Source: iScience. 2021 Nov 9;24(12):103416. doi: 10.1016/j.isci.2021.103416 (PMC8637483; doi:10.1016/j.isci.2021.103416)
Supplement: Document S1. Figures S1–S4 and Table S1 [file mmc1.pdf]

iScience, Volume *24*

Supplemental information

Analyzing gut microbiota composition in individual *Anopheles* mosquitoes after experimental treatment

Aminata Fofana, Mathilde Gendrin, Ottavia Romoli, G. Armel Bienvenu Yarbanga, Georges Anicet Ouédraogo, Rakiswende Serge Yerbanga, and Jean-Bosco Ouédraogo

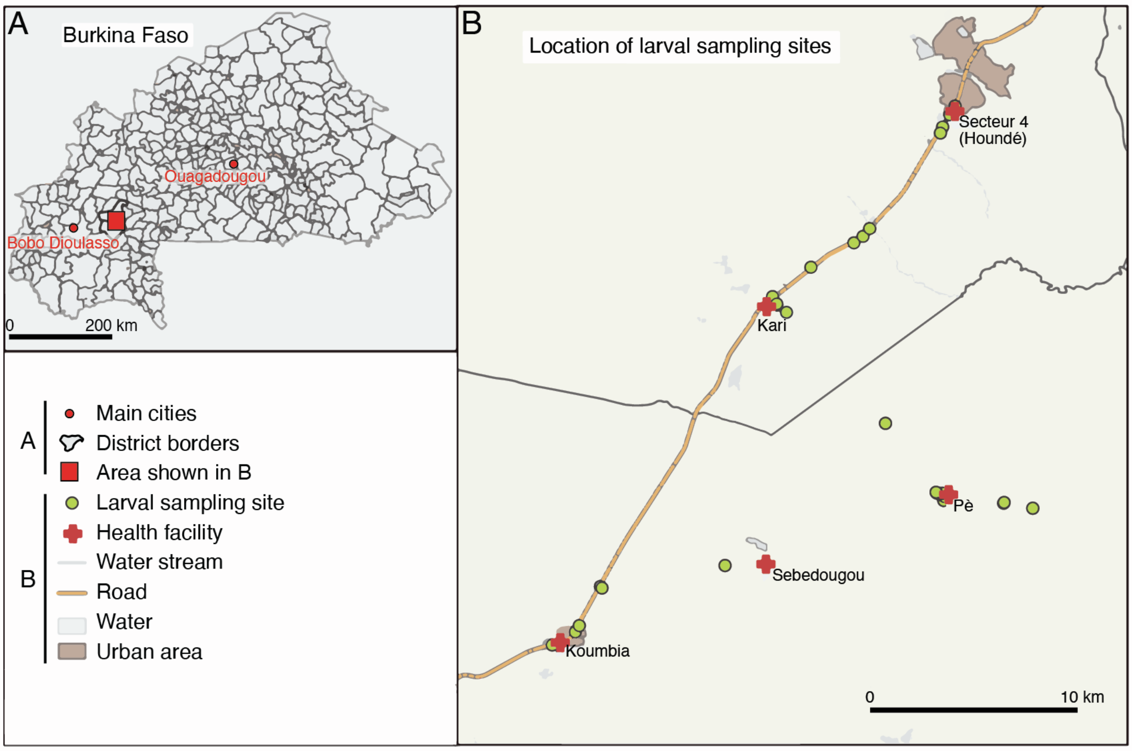

Figure S1. Location of the field sites, related to Table 1. A, Map of the Burkina Faso highlighting in red the location of the study. **B**, Precise map of the larva sampling area within these districts. Maps based on data from (Base Nationale de Données Topographiques du Burkina Faso (BNDT), 2015a, 2015b; Openstreetmap contributors, n.d.)

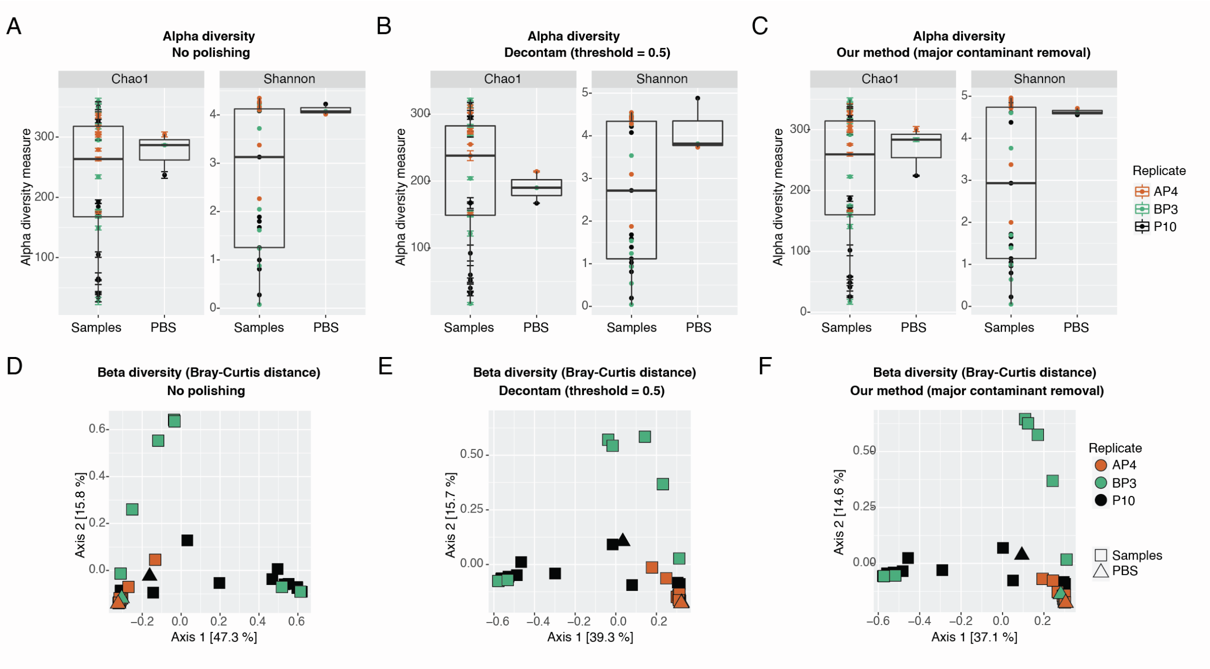

Figure S2. Test of two alternative methods to identify the major contaminant ASVs, related to STAR Methods. A-C, Alpha diversity plots measured with Chao and Shannon indexes of samples and PBS without any polishing (**A**), after removing identified contaminants using the Deconam package (**B**) and with our method (**C**). (**D-F**) Beta diversity of gut bacterial microbiota of *An. gambiae s.l.* mosquitoes represented by a Principal Coordinates Analysis (PCoA) plot of Bray-Curtis dissimilarity following the same three methods. In **A-F**, single points correspond to data from individual mosquitoes. Color-code corresponds to replicates (orange: AP4, no gametocytes; green: BP3, gametocytes; black: P10, gametocytes). In (**A-C**), Wilcoxon test, no polishing: Chao1 index p = 0.76, Shannon index p = 0.35; "decontam": Chao1 index p = 0.76, Shannon index p = 0.23; our method: Chao1 index p = 0.76, Shannon index p = 0.42; analysis performed on non-antibiotic treated samples. In (**D**), PERMANOVA between samples and negative controls, F = 1.7, p = 0.09; in (**E**), PERMANOVA, F = 1.5, p = 0.148; in (**F**) PERMANOVA, F = 1.7, p = 0.045

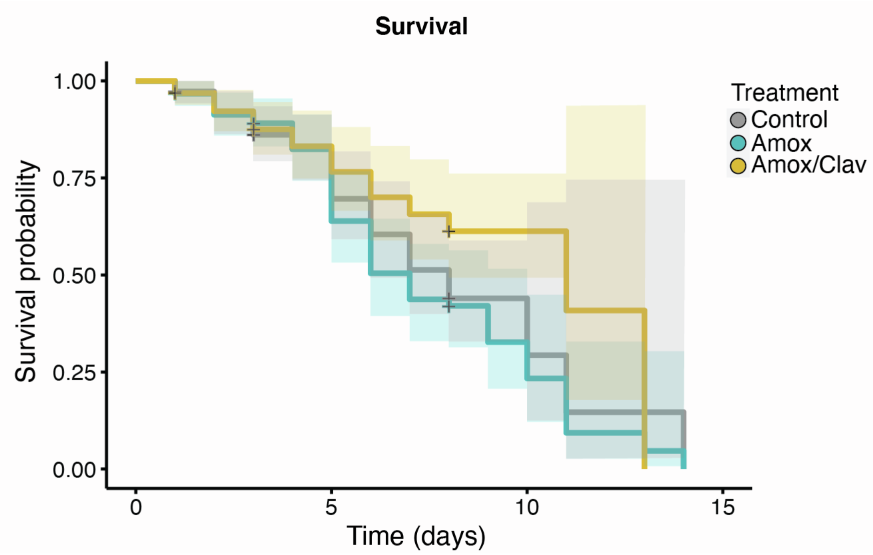

Figure S3. Antibiotic treatment with amoxicillin at high concentration does not significantly affect mosquito survival, related to Figure 3. Survival of the mosquitoes from the day of blood feeding according to blood treatment.

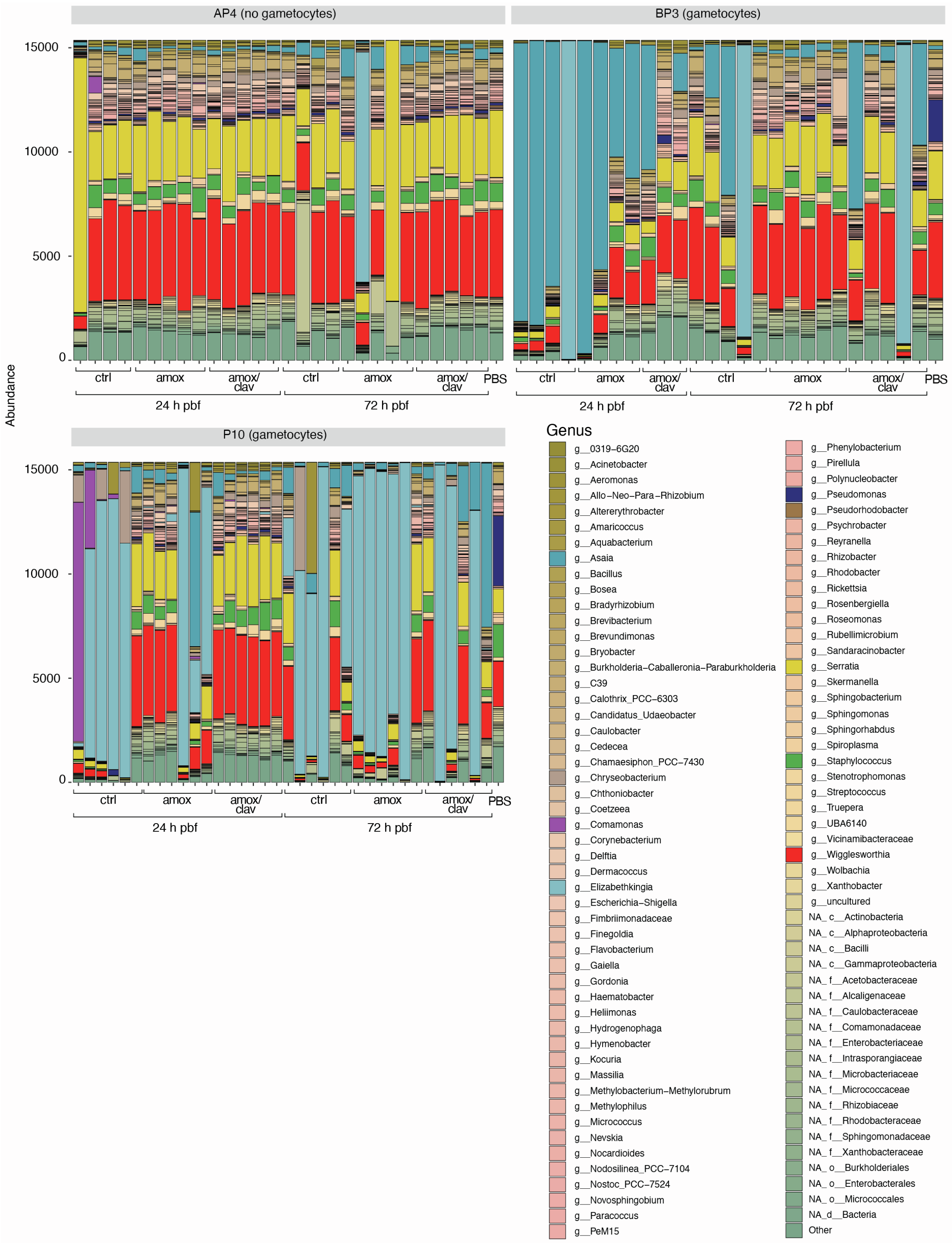

Figure S4. Relative abundance of each detected bacterial genus in each mosquito, related to Figure 4. The nine main genera are indicated in bright colour while secondary genera are indicated in pastel colours. When determination of the genus is impossible (NA), higher taxa were indicated (c: class; f: family; o: order; d: kingdom)

Table S1. Description of the metadata of each sample, related to STAR Methods

Metadata associated to the study: library name (sample-id), code including all relevant information, date of collection, sample identification number, replicate, type of blood feeding (with or without gametocytes), antibiotic treatment, mosquito species and time of collection. BF – blood feeding; rep. – replicate; gameto. – blood containing gametocytes; uninf. – uninfected blood; ND – Non determined species (Samples for which PCR results did not allow any clear species identification).

Sample-id	Code	Date dd_mm_yy	Rep.	Type BF	Treatment	Species	Time post BF
BF262_S1	A-AMOX-CLAV-24-1	25_09_19	BP3	gameto.	amox/clav	*An. gambiae*	24
BF263_S2	A-AMOX-CLAV-24-2	25_09_19	BP3	gameto.	amox/clav	ND	24
BF264_S3	A-AMOX-CLAV-24-3	25_09_19	BP3	gameto.	amox/clav	*An. gambiae*	24
BF265_S4	A-AMOX-24-1	25_09_19	BP3	gameto.	amox	*An. gambiae*	24
BF266_S5	A-AMOX-24-2	25_09_19	BP3	gameto.	amox	ND	24
BF267_S6	A-AMOX-24-3	25_09_19	BP3	gameto.	amox	ND	24
BF268_S7	A-AMOX-24-4	25_09_19	BP3	gameto.	amox	*An. gambiae*	24
BF269_S8	A-CT-24-1	25_09_19	BP3	gameto.	mock ctrl	ND	24
BF270_S9	A-CT-24-2	25_09_19	BP3	gameto.	mock ctrl	ND	24
BF271_S10	A-CT-24-3	25_09_19	BP3	gameto.	mock ctrl	ND	24
BF272_S11	A-CT-24-4	25_09_19	BP3	gameto.	mock ctrl	ND	24
BF273_S12	A-AMOX-CLAV-72-1	25_09_19	BP3	gameto.	amox/clav	*An. gambiae*	72
BF274_S13	A-AMOX-CLAV-72-2	25_09_19	BP3	gameto.	amox/clav	*An. gambiae*	72
BF275_S14	A-AMOX-CLAV-72-3	25_09_19	BP3	gameto.	amox/clav	*An. gambiae*	72
BF276_S15	A-AMOX-CLAV-72-4	25_09_19	BP3	gameto.	amox/clav	ND	72
BF277_S16	A-AMOX-CLAV-72-5	25_09_19	BP3	gameto.	amox/clav	*An. gambiae*	72
BF278_S17	A-AMOX-72-1	25_09_19	BP3	gameto.	amox	*An. coluzzii*	72
BF279_S18	A-AMOX-72-2	25_09_19	BP3	gameto.	amox	*An. gambiae*	72
BF280_S19	A-AMOX-72-3	25_09_19	BP3	gameto.	amox	*An. gambiae*	72
BF281_S20	A-AMOX-72-4	25_09_19	BP3	gameto.	amox	*An. gambiae*	72
BF282_S21	A-AMOX-72-5	25_09_19	BP3	gameto.	amox	*An. gambiae*	72
BF283_S22	A-CT-72-1	25_09_19	BP3	gameto.	mock ctrl	ND	72
BF284_S23	A-CT-72-2	25_09_19	BP3	gameto.	mock ctrl	ND	72
BF285_S24	A-CT-72-3	25_09_19	BP3	gameto.	mock ctrl	ND	72
BF286_S25	A-CT-72-4	25_09_19	BP3	gameto.	mock ctrl	ND	72
BF287_S26	A-CT-72-5	25_09_19	BP3	gameto.	mock ctrl	ND	72
BF418_S27	A-PBS	25_09_19	BP3	PBS	PBS	PBS	PBS
BF419_S28	B-AMOX-CLAV-24-1	14_10_19	AP4	uninf.	amox/clav	*An. gambiae*	24
BF420_S29	B-AMOX-CLAV-24-2	14_10_19	AP4	uninf.	amox/clav	*An. gambiae*	24
BF421_S30	B-AMOX-CLAV-24-3	14_10_19	AP4	uninf.	amox/clav	*An. gambiae*	24
BF422_S31	B-AMOX-CLAV-24-4	14_10_19	AP4	uninf.	amox/clav	*An. gambiae*	24
BF423_S32	B-AMOX-CLAV-24-5	14_10_19	AP4	uninf.	amox/clav	*An. gambiae*	24
BF424_S33	B-AMOX-24-1	14_10_19	AP4	uninf.	amox	*An. arabiensis*	24

Sample-id	Code	Date dd_mm_yy	Rep.	Type BF	Treatment	Species	Time post BF
BF425_S34	B-AMOX-24-2	14_10_19	AP4	uninf.	amox	*An. arabiensis*	24
BF426_S35	B-AMOX-24-3	14_10_19	AP4	uninf.	amox	*An. gambiae*	24
BF427_S36	B-AMOX-24-4	14_10_19	AP4	uninf.	amox	*An. gambiae*	24
BF428_S37	B-AMOX-24-5	14_10_19	AP4	uninf.	amox	*An. gambiae*	24
BF429_S38	B-CT-24-1	14_10_19	AP4	uninf.	mock ctrl	*An. gambiae*	24
BF430_S39	B-CT-24-2	14_10_19	AP4	uninf.	mock ctrl	*An. gambiae*	24
BF431_S40	B-CT-24-3	14_10_19	AP4	uninf.	mock ctrl	*An. gambiae*	24
BF432_S41	B-CT-24-4	14_10_19	AP4	uninf.	mock ctrl	*An. arabiensis*	24
BF513_S42	B-AMOX-CLAV-72-1	14_10_19	AP4	uninf.	amox/clav	*An. gambiae*	72
BF514_S43	B-AMOX-CLAV-72-2	14_10_19	AP4	uninf.	amox/clav	*An. gambiae*	72
BF515_S44	B-AMOX-CLAV-72-3	14_10_19	AP4	uninf.	amox/clav	*An. arabiensis*	72
BF516_S45	B-AMOX-CLAV-72-4	14_10_19	AP4	uninf.	amox/clav	*An. gambiae*	72
BF517_S46	B-AMOX-CLAV-72-5	14_10_19	AP4	uninf.	amox/clav	*An. gambiae*	72
BF533_S47	B-AMOX-72-1	14_10_19	AP4	uninf.	amox	*An. arabiensis*	72
BF534_S48	B-AMOX-72-2	14_10_19	AP4	uninf.	amox	*An. coluzzii*	72
BF535_S49	B-AMOX-72-3	14_10_19	AP4	uninf.	amox	ND	72
BF536_S50	B-AMOX-72-4	14_10_19	AP4	uninf.	amox	*An. gambiae*	72
BF538_S51	B-AMOX-72-5	14_10_19	AP4	uninf.	amox	*An. arabiensis*	72
BF547_S52	B-CT-72-1	14_10_19	AP4	uninf.	mock ctrl	*An. gambiae*	72
BF548_S53	B-CT-72-2	14_10_19	AP4	uninf.	mock ctrl	*An. arabiensis*	72
BF549_S54	B-CT-72-3	14_10_19	AP4	uninf.	mock ctrl	*An. gambiae*	72
BF550_S55	B-CT-72-4	14_10_19	AP4	uninf.	mock ctrl	*An. gambiae*	72
BF551_S56	B-PBS	14_10_19	AP4	PBS	PBS	PBS	PBS
BF1386_S63	C-AMOX-24-1	03_12_19	P10	gameto.	amox	*An. coluzzii*	24
BF1387_S64	C-AMOX-24-2	03_12_19	P10	gameto.	amox	*An. coluzzii*	24
BF1388_S65	C-AMOX-24-3	03_12_19	P10	gameto.	amox	*An. coluzzii*	24
BF1389_S66	C-AMOX-24-4	03_12_19	P10	gameto.	amox	*An. gambiae*	24
BF1390_S67	C-AMOX-24-5	03_12_19	P10	gameto.	amox	*An. gambiae*	24
BF1391_S68	C-AMOX-24-6	03_12_19	P10	gameto.	amox	*An. gambiae*	24
BF1396_S61	C-AMOX-CLAV-24-5	03_12_19	P10	gameto.	amox/clav	*An. gambiae*	24
BF1401_S57	C-AMOX-CLAV-24-1	03_12_19	P10	gameto.	amox/clav	*An. coluzzii*	24
BF1402_S58	C-AMOX-CLAV-24-2	03_12_19	P10	gameto.	amox/clav	*An. coluzzii*	24
BF1403_S59	C-AMOX-CLAV-24-3	03_12_19	P10	gameto.	amox/clav	*An. coluzzii*	24
BF1404_S60	C-AMOX-CLAV-24-4	03_12_19	P10	gameto.	amox/clav	*An. gambiae*	24
BF1405_S62	C-AMOX-CLAV-24-6	03_12_19	P10	gameto.	amox/clav	*An. gambiae*	24

Sample-id	Code	Date dd_mm_yy	Rep.	Type BF	Treatment	Species	Time post BF
BF1407_S69	C-CT-24-1	03_12_19	P10	gameto.	mock ctrl	*An. gambiae*	24
BF1410f_S70	C-CT-24-3	03_12_19	P10	gameto.	mock ctrl	*An. gambiae*	24
BF1410a_S71	C-CT-24-2	03_12_19	P10	gameto.	mock ctrl	*An. gambiae*	24
BF1411f_S72	C-CT-24-5	03_12_19	P10	gameto.	mock ctrl	*An. coluzzii*	24
BF1411a_S73	C-CT-24-4	03_12_19	P10	gameto.	mock ctrl	*An. coluzzii*	24
BF1413_S74	C-CT-24-6	03_12_19	P10	gameto.	mock ctrl	*An. coluzzii*	24
BF1419_S81	C-AMOX-72-1	03_12_19	P10	gameto.	amox	*An. gambiae*	72
BF1420_S84	C-AMOX-72-4	03_12_19	P10	gameto.	amox	*An. arabiensis*	72
BF1421_S85	C-AMOX-72-5	03_12_19	P10	gameto.	amox	*An. arabiensis*	72
BF1422_S82	C-AMOX-72-2	03_12_19	P10	gameto.	amox	*An. gambiae*	72
BF1423_S86	C-AMOX-72-6	03_12_19	P10	gameto.	amox	*An. arabiensis*	72
BF1427_S83	C-AMOX-72-3	03_12_19	P10	gameto.	amox	*An. gambiae*	72
BF1429_S75	C-AMOX-CLAV-72-1	03_12_19	P10	gameto.	amox/clav	*An. gambiae*	72
BF1430_S76	C-AMOX-CLAV-72-2	03_12_19	P10	gameto.	amox/clav	*An. gambiae*	72
BF1432_S78	C-AMOX-CLAV-72-4	03_12_19	P10	gameto.	amox/clav	*An. arabiensis*	72
BF1434_S79	C-AMOX-CLAV-72-5	03_12_19	P10	gameto.	amox/clav	*An. arabiensis*	72
BF1435_S80	C-AMOX-CLAV-72-6	03_12_19	P10	gameto.	amox/clav	*An. arabiensis*	72
BF1438_S77	C-AMOX-CLAV-72-3	03_12_19	P10	gameto.	amox/clav	*An. gambiae*	72
BF1442_S92	C-CT-72-6	03_12_19	P10	gameto.	mock ctrl	*An. arabiensis*	72
BF1443_S87	C-CT-72-1	03_12_19	P10	gameto.	mock ctrl	*An. gambiae*	72
BF1444f_S88	C-CT-72-3	03_12_19	P10	gameto.	mock ctrl	*An. gambiae*	72
BF1444a_S89	C-CT-72-2	03_12_19	P10	gameto.	mock ctrl	*An. gambiae*	72
BF1445f_S90	C-CT-72-5	03_12_19	P10	gameto.	mock ctrl	*An. arabiensis*	72
BF1445a_S91	C-CT-72-4	03_12_19	P10	gameto.	mock ctrl	*An. arabiensis*	72
BF1443_S93	C-PBS	03_12_19	P10	PBS	PBS	PBS	PBS
